# Successful School‐to‐Training Transitions—Can Individual Goal‐Striving Resources Compensate for Structural Obstacles in the Local Context?

**DOI:** 10.1002/jad.70037

**Published:** 2025-08-12

**Authors:** Nele Theuer, Katarina Weßling

**Affiliations:** ^1^ Federal Institute for Vocational Education and Training (BIBB) Bonn Germany; ^2^ Faculty of Economics and Social Sciences University of Tübingen Tübingen Germany; ^3^ Research Centre for Education and the Labour Market (ROA) Maastricht University Maastricht Netherlands

**Keywords:** flexible goal adjustment, goal‐striving resources, school‐to‐training‐transition, structural obstacles, tenacious goal pursuit, vocational education and training

## Abstract

**Introduction:**

While it is well‐established that structural obstacles such as low local employment opportunities negatively affect adolescents’ school‐to‐work transitions, the impact of individual agency in relation to these obstacles is understudied. Hence, we focus on the key research question of how adolescents’ goal‐striving resources—tenacious goal pursuit (TGP) and flexible goal adjustment (FGA)—affect successful transitions from secondary school to vocational education and training (VET) against the backdrop of structural obstacles in the local context.

**Methods:**

We measured transition success in terms of a) the start of a VET position, b) concordance between attained and aspired VET position, c) deviance from the aspired position and d) satisfaction with the attained VET position. We examined our research questions in a longitudinal design, using data from the German National Educational Panel Study (NEPS). Our sample consisted of adolescents who graduated in 2012/2016 with the aspiration to start VET (*N* = 3382; *M*_Age_ = 16.77; *n*_Females_ = 1524). We conducted multiple regression analyses to answer our research questions.

**Results:**

We found interaction effects of TGP × FGA on three out of four indicators of transition success. Their direction did not only depend on the outcome variable under examination but also on the structure of the local context: High levels of both TGP and FGA are helpful in favorable contexts—that means when local unemployment is low—but maladaptive when structural obstacles are high.

**Conclusion:**

We recommend that support programs for adolescents that aim to enhance goal‐striving resources should consider these complex interrelations.

## 1 Introduction

In countries with well‐developed vocational education and training (VET) systems such as Germany, VET represents the main educational route towards an occupational qualification (Bundesinstitut für Berufsbildung 2023). Transitioning from school to VET marks the initial step of an individual's professional career and is highly consequential for adolescents’ career paths: Smooth transitions are associated with stable careers (Heckhausen and Tomasik 2002; Hillmert et al. 2017; Walden 2021), whereas delayed and discontinuous transitions increase the risk of unstable career trajectories, characterized by periods of unemployment or low‐qualified jobs (Achatz et al. 2022; Brzinsky‐Fay 2022; Brzinsky‐Fay and Solga 2016).

Hence, the school‐to‐training transition can be considered an important developmental goal in adolescence (Basler and Kriesi 2019). Accordingly, many researchers have analyzed these transitions, either by examining the successful attainment of a VET position or by focusing on subjective measures of transition success—such as the mismatch between aspired and attained VET position (Hillmert et al. 2017; Nießen et al. 2020; Schels et al. 2022). Surprisingly however, only few studies have taken a *goal‐striving perspective*; those that do only focus on the impact of specific individual strategies and not of the full range of goal‐striving resources (Haase et al. 2008; Heckhausen and Tomasik 2002). Hence, knowledge of how goal‐striving can help or hinder the school‐to‐VET transition remains limited. We will add to this study area by pursuing two innovative objectives.

Firstly, we aim to provide a comprehensive understanding of how two broad individual goal‐striving resources support adolescents during the school‐to‐VET transition by applying Brandtstädter and Renner's (1990) well‐established *dual‐process framework of goal pursuit and goal adjustment*.

Secondly, we will examine how the usefulness of these resources depends on other influential factors. Goal‐striving theory stresses the dependency of goal‐striving effects on conditions of an individual's environment (Brandtstädter and Rothermund 2002; Mele et al. 2023). In regard to the school‐to‐VET transition, structural obstacles such as high unemployment or low availability of training places have been identified as risk factors for a successful transition (Busse 2020; Hillmert et al. 2017; Weßling et al. 2015). Thus, we will explore how the usefulness of individual goal‐striving resources is hindered or enhanced by such structural obstacles.

Taken together, our study contributes to research on the usefulness of different goal‐striving resources during the school‐to‐training transition under varying structural conditions. Thereby we aim to enhance knowledge of how adolescents can be best supported during this developmental task.

### 1.1 Dual‐Process Framework of Tenacious Goal Pursuit and Flexible Goal Adjustment

Brandtstädter's dual‐process framework defines individuals as *active agents* who are *embedded in contexts* and understands development as an iterative process of balancing out individually set goals and structural obstacles (Brandtstädter 2006; Brandtstädter and Baltes‐Götz 1990). The framework identifies two modes of coping with such developmental processes: assimilation and accommodation (Brandtstädter and Renner 1990; Brandtstädter and Rothermund 2002).

Assimilative strategies describe goal‐pursuit behaviors that aim at adapting a given situation to the set goal (e.g., instrumental actions), whereas accommodative strategies serve to adapt individual goals to structural obstacles (e.g., goal adjustment). In the dual‐process model, Brandtstädter and colleagues identify two stable resources that refer to an individuals’ readiness to make use of either accommodation or assimilation during goal‐striving: *tenacious goal pursuit* (TGP) and *flexible goal adjustment* (FGA). TGP describes the tendency to use assimilative strategies and FGA that to use accommodative ones (Brandtstädter 2015, 2006; Brandtstädter and Rothermund 2002). Individuals can be high (or low) in both TGP and FGA, as they are independent from each other (Brandtstädter and Renner 1990; Brandtstädter and Rothermund 2002).

#### 1.1.1 Effects of Goal‐Striving Resources

The positive effect of TGP (i.e., the default use of assimilative goal‐strategies) on subjective and objective indicators of developmental success has been well‐established (e.g., Haratsis et al. 2016; Siltanen et al. 2019; Zhang 2020). Furthermore, goal‐striving strategies closely linked to TGP (Brandtstädter and Rothermund 2002; Määttä et al. 2002) are positively associated with school‐to‐VET‐transition success (Haase et al. 2008; Holtmann et al. 2017; Neuenschwander et al. 2024).

Likewise, a large body of research has consistently presented positive effects of FGA (i.e., the default use of accommodative strategies) on indicators of well‐being and goal attainment (e.g., Kelly et al. 2013; Sahdra et al. 2022; van Diemen 2020). In the context of VET, studies show that accommodative behavior positively affects the transition to VET (Heckhausen and Tomasik 2002; Tomasik et al. 2009).

As the dual‐process framework does not explicitly specify how TGP and FGA interact, two contradictory theories have emerged regarding their joint impact: *The Regulatory Dilemma Hypothesis (RDH)* states that when people are highly tenacious and flexible, they might have difficulty choosing a goal‐striving strategy, which results in a maladaptive state of inactivity (i.e., negative interaction; Bak and Brandtstädter 1998; Brandtstädter 2015; Sahdra et al. 2022). In contrast, *the Complementary Hypothesis (CH)* suggests that these people can adaptively choose from the whole range of strategies (i.e. assimilative and accommodative), which results in higher situation‐strategy‐fit (i.e., positive interaction; Bailly et al. 2016; Sahdra et al. 2022). Since empirical evidence exists for both theories (e.g., Arewasikporn et al. 2019; Coffey et al. 2014; Heyl et al. 2007; Sahdra et al. 2022), we expect to find either of these interaction effects.

Accordingly, we hypothesize:

We will find a positive effect of TGP on the school‐to‐VET transition.

We will find a positive effect of FGA on the school‐to‐VET transition.

We will find either a positive (CH) or a negative interaction effect (RDH) of FGA and TGP on the school‐to‐VET transition.

#### 1.1.2 Moderation by Structural Obstacles

The dual‐process framework predicts the usefulness of TGP and FGA to vary depending on (structural) circumstances (Brandtstädter and Renner 1990; Brandtstädter and Rothermund 2002). This understanding that different structural or institutional contexts call for varying strategies has since been established in empirical research on goal‐striving behavior (Brandstätter and Bernecker 2022; Heckhausen and Wrosch 2022; Schoon and Heckhausen 2019; Schoon and Lyons‐Amos 2017). Studies show that active goal pursuit (i.e., assimilation) can become a waste of effort when structural obstacles are so high that goal achievement is highly unlikely, whereas goal adjustment (i.e., accommodation) is especially helpful in such circumstances (Heckhausen and Wrosch 2022). Accordingly, the dual‐process model predicts that structural obstacles moderate TGP and FGA effects differently, resulting in a positive interaction of obstacles with FGA and a negative interaction with TGP (Brandtstädter 2015; Brandtstädter and Greve 1994; Brandtstädter and Rothermund 2002).

Many studies provide evidence for the suggested moderation effects, although influences on FGA are found more consistently (Coffey et al. 2014; Heyl et al. 2007). Nevertheless, empirical evidence also supports the moderation effect on TGP, showing that TGP effects on development—including transition success—are stronger when obstacles are low (Pals and Kaplan 2013; Schoon and Lyons‐Amos 2017; Wettstein et al. 2019). Flexible goal‐striving was found to be more successful in unfavorable circumstances (Coffey et al. 2014; Hamm et al. 2022; Heyl et al. 2007; Wettstein et al. 2019).

A question that so far remained understudied in goal‐striving research is how the TGP×FGA interaction depends on structural obstacles. Two mechanisms are plausible: Some literature suggests that when obstacles are high, the access to a wide range of strategies might be most helpful (Mayo 2024; Zimmer‐Gembeck 2021). That implies that a positive TGP × FGA interaction would become larger under difficult contextual circumstances. Alternatively, it could be argued that when contextual obstacles are high, TGP loses its usefulness while FGA gains usefulness (Brandstätter and Bernecker 2022; Heckhausen and Wrosch 2022). This would result in a negative TGP × FGA interaction which becomes larger under unfavorable structural circumstances. Based on these considerations, we hypothesize:

We will find a negative interaction of TGP and structural obstacles on the school‐to‐VET‐transition.

We will find a positive interaction effect of FGA and structural obstacles on the school‐to‐VET‐transition.

We will find that structural obstacles moderate the TGP×FGA interaction on the school‐to‐VET transition, specifically that large structural obstacles either lead to a larger positive or a larger negative TGP×FGA interaction.

### 1.2 The German Case

To test our hypotheses we make use of the German educational system, where adolescents graduate from secondary school with lower (*Hauptschulabschluss*) or intermediate leaving certificates (*Realschulabschluss*) or higher education entrance degrees *((Fach‐)Hochschulreife*)—after grade 9, 10 or 12/13 respectively. After graduation, students from all tracks are qualified to enter vocational training, although especially those without higher education entrance degrees chose this pathway (Döbert 2015). Consequently, competition to obtain a (desired) VET occupation is high amongst graduates. We believe that in this setting individual resources—such as TGP and FGA—significantly impact adolescents’ transition chances.

Moreover, we believe that certain structural characteristics of the German VET system influence the usefulness of such strategies. We argue that the chance to obtain a (desired) VET occupation is highly dependent on labor‐market conditions: The German VET system offers two types of training, dual and school‐based VET. Dual VET occupations are taught predominantly in VET firms, while shorter periods are spent in VET schools. Access to dual VET occupations is not institutionally regulated; instead, VET firms decide whether and whom they hire as apprentices (Solga et al. 2014). This structure, in combination with the limited regional mobility of adolescents (Eckelt and Schauer 2019; Solga et al. 2014), has the effect that companies are less willing and able to offer VET when local labor‐markets perform poorly. Consequently, competition among adolescents in these contexts intensifies (Eckelt and Schauer 2019; Hillmert et al. 2017; Troltsch and Walden 2010).

School‐based VET occupations are mostly taught at VET schools in combination with intervals of workplace experience mostly in the form of internships. Access to school‐based VET is regulated by the German federal states, for instance regarding required school‐leaving certificates (Solga et al. 2014). Although not directly influenced by poor labor‐market conditions, they diminish the possibility to enter school‐based VET indirectly as they lead to a shift of adolescents’ interest from dual VET to school‐based VET, also resulting in higher competition within this system (Kleinert and Jacob 2012).

We believe that by focusing on the German educational system and local labor‐markets we are able to explore how goal‐striving resources influence school‐to‐training transitions and how these effects are moderated by structural obstacles.

## 2 Material and Methods

### 2.1 Sample

We used data from Starting Cohorts 3 (SC3) and 4 (SC4) of the German National Educational Panel Study (NEPS; Blossfeld and Roßbach 2019; NEPS Network 2023a, 2023b)^1^. NEPS is a multi‐cohort‐sequence survey that follows adolescents’ educational and occupational trajectories over their life course. It provides representative samples of 5th (SC3) and 9th (SC4) grade students based on a stratified multistage sampling strategy, which means that samples are drawn separately in each school type. Within school types, schools and then classrooms are selected randomly (Aßmann et al. 2011). The survey provides a large set of information on students, their educational careers, and their school and home environment, allowing us to examine the assumed effects in a longitudinal design (Blossfeld et al. 2011; Fuß et al. 2016; Roßbach and von Maurice 2018).

We worked with a sample of adolescents who graduated after grade 10 (SC4: summer 2012; SC3: summer 2016) and had voiced the aspiration to start VET. This leaves us with a sample of *N* = 3,382 successful graduates. School leavers without any degree or from special‐needs schools were not included in the sample because they often do not fulfill the requirements to enroll for school‐based VET (Kleinert and Jacob 2012; Solga et al. 2014).

Predictor variables were measured during the last year of school, which we assume to be the most meaningful for the transition process (Bundesagentur für Arbeit BA 2024).

### 2.2 Focal Measures

#### 2.2.1 Dependent Variables: Successful Transition to VET

We used *four indicators* to measure both objective and subjective aspects of transition success. All of these have been used in previous research and hence can be considered standard measures of the school‐to‐VET transition (Hartung and Weßling 2024; Nießen et al. 2020, 2022; Schels et al. 2022; Wicht et al. 2024).

We examined whether adolescents have successfully started a VET position 1 year after graduation (*DV1_start*). The transition was considered successful if adolescents had been in VET for at least 5 months, excluding those who had dropped out of VET during the first few months (Baas and Philipps 2017; Beicht and Walden 2013). We dummy‐coded it accordingly. For those who successfully started VET, we analyzed three more variables of transition success.

Firstly, we examined the concordance between the attained and the aspired VET position (*DV2_concordance*). Students rated the following item on a five‐point scale from *does not apply at all* (1) to *does completely apply* (5): *This profession is my desired profession*. This question was answered mostly in autumn/winter directly after graduation or 1 year later. In addition to this *self‐reported* measure of concordance, we also measured the *objective* deviance of attained and aspired VET position *(DV3_deviance*; Wicht et al. 2024 *)*. To do so, we obtained occupational groups of both the aspired and the attained VET position using two‐digit codes of the German Classification of Occupations (KldB‐2010; Bundesagentur für Arbeit 2010; Paulus and Matthes 2013). The former was collected within the year before graduation, the latter when adolescents first reported their VET position in the survey. We then subtracted the code for the attained VET position from that of the aspired position and classified any deviance from zero as 1 (Schels et al. 2022).

Lastly, we measured satisfaction with the attained VET position *(DV4_satisfaction)* using the following item: *How satisfied are you with your vocational training?* The response scale ranged from *completely dissatisfied* (0) *to completely satisfied* (10). It was measured 1 year after graduation (Nießen et al. 2020).

#### 2.2.2 Independent Variables: Tenacious Goal Pursuit and Flexible Goal Adjustment

TGP and FGA were measured with short versions of the *Tenacious Goal Pursuit and Flexible Goal Adjustment Scales* by Brandtstädter and Renner (1990) in autumn/winter before graduation from secondary school. As recommended by Sahdra et al. (2022) and based on results from our own confirmatory factor analyses (Appendix A), we worked with three‐item‐versions of each scale (Table 1).

**Table 1:** Items of the tenacious goal pursuit and flexible goal adjustment scales.

Scale	Items
Tenacious Goal Pursuit	The more difficult a goal is to achieve, the more desirable it often seems to me
	I tend to fight even in hopeless situations
	Once I have set my mind on something, I will not be discouraged even by great difficulties.
Flexible Goal Adjustment	Even if something goes wrong, I do see some progress somewhere
	I can also gain something from giving up on my goals
	I can also find a good side in the unpleasant things in life

Response scales ranged from *does not apply at all* (1) to *does completely apply (5)*. Considering the shortness of the scales (Stanley and Edwards 2016), reliability is acceptable (α_TGP_ = 0.55; α_FGA_ = 0.57). For each construct we calculated sum scores (Siltanen et al. 2019; Tourunen et al. 2020). As a robustness analysis, we calculated all models using the five‐item versions of both scales. Results did not change substantially (Appendix A).

#### 2.2.3 Moderator Variable: Structural Obstacles

**Figure 1:**
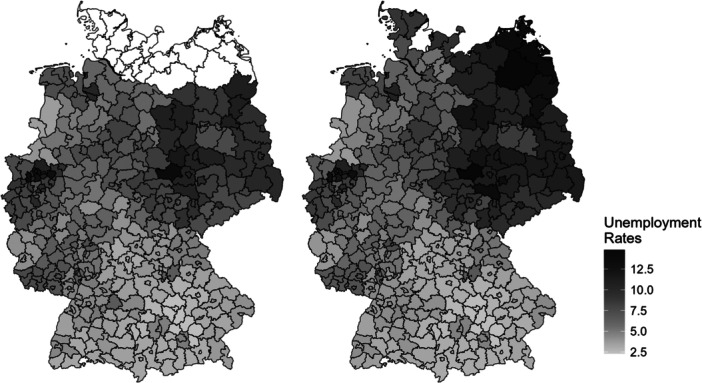
Local Unemployment Rates. Notes. Own depiction. Left: unemployment rates for SC3 (2015); Right: unemployment rates for SC3 (2011). Local units are administrative district (NUTS‐3 regions). Missing data is depicted in white. Data: Federal Employment Agency (Bundesagentur für Arbeit 2012, 2016) and Federal Agency for Cartography and Geodesy (GeoBasis‐DE 2014).

To measure structural obstacles, we used local unemployment rates as it is the strongest predictor for the socioeconomic conditions in a local context (Hillmert et al. 2017; Weßling and Bechler 2019) and highly correlated with the local provision of VET places (Glauser and Becker 2016; Hartung et al. 2022; Zwysen 2016) (Figure 1).

The data stems from the Federal Employment Agency and provides information about unemployment rates based on all civilian employees in employment agency districts (Bundesagentur für Arbeit 2012, 2016). Since employment agency districts are the local areas in which adolescents typically search for training, they provide a meaningful mobility radius to analyze the school‐to‐VET‐transition (Eckelt and Schauer 2019; Heineck et al. 2011; Weßling et al. 2015). We used annual averages from 2011 (SC4) and 2015 (SC3). We linked this information to the NEPS data by adolescents’ home districts and—if not available—by school districts. School and home district are the same in most cases.

#### 2.2.4 Covariates

We systematically included covariates, which potentially confound the relationship between our predictor variables and transition success (Beicht and Walden 2019; Nießen et al. 2022; Weßling et al. 2015).

Information regarding *sex* and *migration background* was collected at multiple time points and was dummy coded. Migration background was coded as 1 if at least one parent was born in a foreign country (Hillmert and Weßling 2014; Olczyk et al. 2014).

Moreover, we controlled for *school leaving degree* and *final grade* on the corresponding certificate. We dummy‐coded information on whether people in our sample obtained a lower or intermediate school leaving degree. Final grades can range from 1 (best) to 6 (worst).

To control for *socioeconomic status*, we used parental HISEIs (Ganzeboom 2010; Highest International Socioeconomic Index of Occupational Status, ISEI‐08; Ganzeboom et al. 1992; Holtmann et al. 2017). We worked with the most recent information collected from parent questionnaires and—if not available—filled it in with student questionnaire data.

Lastly, we created dummy‐coded covariates for the *aspired type of VET* within 1 year before graduation (school‐based vs. dual VET) as well as for *starting cohort* (SC3 vs. SC4).

## 3 Results

If not stated otherwise, analyses were conducted using R after multiple imputation of missing data with the *mice* package (R Core Team 2023; Buuren and Groothuis‐Oudshoorn 2011). We included model variables, interaction terms and auxiliary variables into the imputation model (50 imputations with 20 iterations). Results were pooled using Rubin's Rules (Enders 2022; Rubin 1987; van Buuren 2018).

### 3.1 Descriptive Analyses

Sample distributions show that most adolescents successfully transition to VET after school (DV1_start), that attained VET positions mostly match adolescents’ aspired occupations and that they are associated with high satisfaction (Table 2).

**Table 2:** Descriptive statistics of focal variables.

Variable		*Mean (SD)*	*N*
TGP		10.84 (1.92)	3382
FGA		9.62 (1.98)	3382
Unemployment rates		6.60 (2.92)	3382
DV2_concordance		4.34 (0.85)	2018
DV4_satisfaction		8.16 (1.48)	2018
SES		44.62 (18.25)	3382
Final grade		2.61 (0.54)	3382
Age		16.77 (0.71)	3382

Variable		Count (Percentage)	*N*
DV1_start	no (0)	860 (25.4%)	3382
	yes (1)	2522 (74.6%)	
DV3_deviance	no (0)	1255 (62.2%)	2018
	yes (1)	763 (37.8%)	
Sex	male (0)	1858 (54.9%)	3382
	female (1)	1524 (45.1%)	
Migration background	no (0)	2708 (80.1%)	3382
	yes (1)	674 (19.0%)	
Leaving certificate	lower (0)	603 (17.8%)	3382
	intermediate (1)	2779 (82.2%)	
Wave	SC4 (0)	2587 (76.5%)	3382
	SC3 (1)	795 (23.5%)	
Aspired type of VET	school‐based VET (0)	967 (28.6%)	3382
	dual VET (1)	2415 (71.4%)	

*Note:* For DV2_concordance, DV3_deviance, and DV4_satisfaction we only included adolescents who successfully started VET (i.e., DV1_start = 1) according to all 50 imputations.

These findings are in line with previous research (Bundesinstitut für Berufsbildung 2013, 2017; Heinecke et al. 2023) and hence speak to the representativity of our sample. Moreover, correlations of covariates and the moderator with outcome variables mostly match well‐established associations between individual or structural circumstances and transition chances, indicating validity of our transition indicators (Weßling et al. 2015).

Low to medium correlations between the outcome variables (except DV1_start) suggest that the four indicators of transition success depict distinct aspects of success and thus support our decision to focus on multiple outcomes (Table 3).

**Table 3:** Correlations of focal variables.

Variable	2	3	4	5	6	7	8	9	10	11	12	13	14	Age
**1. TGP**	0.36***	0.04*	0.03	0.05**	0.03	0.06**	−0.04*	0.03+	−0.04+	−0.02	−0.08***	−0.05**	0.07***	0.05**
**2. FGA**		0.04*	−0.01	0.04*	0.01	0.05*	−0.03	0.08***	0.01	−0.06**	−0.01	−0.003	0.03+	0.04*
**3. Unemployment**			−0.05**	0.03	0.02	−0.01	−0.01	−0.01	−0.05**	−0.1***	0.07***	−0.08***	−0.08***	0.04*
**4. DV1_start**				−0.02	0.03	−0.005	−0.15***	−0.23***	−0.01	0.25***	−0.12***	−0.04+	0.61***	−0.02
**5. DV2_concordance**					−0.2***	0.27***	−0.03+	−0.08***	−0.02	0.04+	−0.02	0.04*	−0.01	−0.02
**6. DV3_deviance**						−0.04+	−0.05*	0.05+	0.02	−0.07*	0.10***	−0.31***	0.06*	0.05*
**7. DV4_satisfaction**							−0.05**	−0.05*	−0.01	0.01	−0.02	−0.002	0.01	−0.05**
8. Sex (0 = male)								0.07***	−0.08***	0.07***	−0.1***	−0.02	−0.44***	−0.03*
9. Migration background									−0.17***	−0.28***	0.09***	−0.12***	−0.13***	0.14***
10. SES										0.14***	−0.05**	0.05*	−0.05*	−0.07***
11. Leaving certificate (0 = lower)											−0.16***	0.09***	0.03*	−0.12***
12. Final grade												−0.04*	−0.06***	0.07***
13. Wave (0 = SC4)													0.04*	−0.12***
14. Aspired type of VET (0 = school‐based VET)														0.04*

*Note:* Tetrachoric correlations were calculated between two binary variables; Pearson correlations between all other variables (Makowski et al. 2022; Revelle 2022).

*** *p* < 0.001

** *p* < 0.01

* *p* < 0.05,

^+^ *p* < 0.10

Lastly, we found a positive correlation between TGP and FGA, which—despite the theoretical independence of the constructs (Brandtstädter and Renner 1990)—is in line with previous studies (Brands et al. 2015; Kelly et al. 2013; Sahdra et al. 2022).

### 3.2 Inferential Analysis

We conducted multiple logistic regression (DV1_start and DV3_deviance) and multiple linear regression (DV2_concordance and DV4_satisfaction) analyses (Nguyen 2020). As robustness checks, we calculated multilevel models (L1: individuals, L2: schools) and found that results did not change substantially (Appendix B).

We z‐standardized numeric variables and used robust standard errors in all models (Hayes and Cai 2007; Tabachnik and Fidell 2014). Regression models were built in a stepwise manner, starting with a covariate model and successively adding all main and interaction effects.

#### 3.2.1 The Effect of Goal‐Striving Resources on the School‐to‐Training Transition

To test the impact of goal‐striving resources on the school‐to‐VET transition we examined three hypotheses, regarding main effects of TGP and FGA as well as their interaction effect on four indicators of VET success (Tables 4, 5, 6, 7).

**Table 4:** Multiple regression model: Effects of TGP, FGA and structural obstacles on start of a VET position (DV1_start).

Predictor	Model 1				Model 2				Model 3				Model 4				Model 5			
	*β*	*SE*	*OR*	*p*	*β*	*SE*	*OR*	*p*	*β*	*SE*	*OR*	*p*	*β*	*SE*	*OR*	*p*	*β*	*SE*	*OR*	*p*
Intercept	−0.49**	0.16	0.61	0.002	−0.48**	0.16	0.62	0.003	−0.46**	0.16	0.63	0.004	−0.46**	0.16	0.63	0.004	−0.47**	0.16	0.62	0.003
Leaving certificate	0.71***	0.14	2.03	< 0.001	0.70***	0.14	2.01	< 0.001	0.70***	0.14	2.02	< 0.001	0.71***	0.14	2.03	< 0.001	0.71***	0.14	2.04	< 0.001
Aspired type of VET	1.89***	0.11	6.59	< 0.001	1.88***	0.11	6.58	< 0.001	1.89***	0.11	6.60	< 0.001	1.89***	0.11	6.60	< 0.001	1.89***	0.11	6.63	< 0.001
Wave	−0.25*	0.12	0.78	.03	−0.26*	0.12	0.77	0.03	−0.25*	0.12	0.78	0.03	−0.25*	0.12	0.78	0.03	−0.26*	0.12	0.77	0.03
Migration background	−0.55***	0.13	0.58	< 0.001	−0.55***	0.13	0.58	< 0.001	−0.54***	0.13	0.58	< 0.001	−0.55***	0.13	0.58	< 0.001	−0.54***	0.13	0.58	< 0.001
Final grade	−0.21***	0.05	0.81	< 0.001	−0.21***	0.05	0.81	< 0.001	−0.21***	0.05	0.81	< 0.001	−0.20***	0.05	0.81	< 0.001	−0.21***	0.05	0.81	< 0.001
SES	−0.08	0.05	0.92	0.15	−0.08	0.05	0.92	0.14	−0.08	0.05	0.92	0.15	−0.08	0.05	0.92	0.14	−0.08	0.05	0.92	0.15
Sex	0.03	0.11	1.03	0.79	0.03	0.11	1.03	0.82	0.02	0.11	1.02	0.82	0.02	0.11	1.02	0.84	0.02	0.11	1.02	0.84
**Unemployment**					**−0.03**	**0.05**	**0.97**	**0.54**	**−0.03**	**0.05**	**0.97**	**0.55**	**−0.03**	**0.05**	**0.97**	**0.54**	**−0.00**	**0.05**	**1.00**	**0.94**
**TGP**					**−0.01**	**0.06**	**0.99**	**0.88**	**−0.02**	**0.06**	**0.98**	**0.78**	**−0.01**	**0.06**	**0.99**	**0.81**	**−0.01**	**0.06**	**0.99**	**0.84**
**FGA**					**−0.02**	**0.05**	**0.98**	**0.69**	**−0.02**	**0.05**	**0.98**	**0.73**	**−0.02**	**0.05**	**0.98**	**0.71**	**−0.02**	**0.05**	**0.98**	**0.76**
**TGP × FGA**									**−0.06**	**0.04**	**0.95**	**0.19**	**−0.06**	**0.04**	**0.95**	**0.19**	**−0.05**	**0.04**	**0.95**	**0.25**
**FGA × unemployment**													**0.04**	**0.05**	**1.04**	**0.49**	**0.04**	**0.05**	**1.04**	**0.44**
**TGP × unemployment**													**−0.04**	**0.05**	**0.96**	**0.44**	**−0.05**	**0.05**	**0.95**	**0.36**
**TGP × FGA × unemployment**																	**−0.06** +	**0.04**	**0.94**	**0.097**
df	7, 5560.75				3, 2858.98				1, 777.35				2, 2363.88				1, 1585.32			
*F*‐value	55.04 (*p* < 0.001)***				0.22 (*p* = 0.88)				1.74 (*p* = 0.19)				0.39 (*p* = 0.67)				2.76 (*p* = 0.097)+			
Pseudo R²	0.16				0.16				0.16				0.16				0.16			
Adjusted Pseudo R²	0.15				0.15				0.15				0.15				0.15			

*** *p* < 0.001

** *p* < 0.01

* *p* < 0.05,

^+^ *p* < 0.10

**Table 5:** Multiple regression models: Effects on concordance between aspired and attained VET position (DV2_concordance).

Predictor	Model 1			Model 2			Model 3			Model 4			Model 5		
	*β*	*SE*	*p*	*β*	*SE*	*p*	*β*	*SE*	*p*	*β*	*SE*	*p*	*β*	*SE*	*p*
Intercept	−0.01	0.10	0.90	−0.03	0.10	0.77	−0.04	0.10	0.69	−0.04	0.10	0.68	−0.05	0.10	0.66
Leaving certificate	0.16*	0.08	0.04	0.18*	0.08	0.03	0.18*	0.08	0.02	0.18*	0.08	0.02	0.18*	0.08	0.02
Aspired type of VET	−0.07	0.07	0.28	−0.07	0.07	0.32	−0.07	0.07	0.30	−0.07	0.07	0.29	−0.07	0.07	0.31
Wave	0.08	0.05	0.13	0.09	0.05	0.10	0.08	0.05	0.11	0.09	0.05	0.11	0.09	0.05	0.11
Migration background	−0.25***	0.07	< 0.001	−0.26***	0.07	< 0.001	−0.26***	0.07	< 0.001	−0.26***	0.07	< 0.001	−0.26***	0.07	< 0.001
Final grade	−0.03	0.02	0.27	−0.02	0.02	0.40	−0.02	0.02	0.38	−0.02	0.02	0.38	−0.02	0.02	0.36
SES	−0.05+	0.03	0.07	−0.05+	0.03	0.07	−0.04+	0.03	0.08	−0.05+	0.03	0.08	−0.04+	0.03	0.08
Sex	−0.13*	0.05	0.01	−0.12*	0.05	0.01	−0.12*	0.05	0.01	−0.12*	0.05	0.01	−0.12*	0.05	0.01
**Unemployment**				**0.02**	**0.02**	**0.33**	**0.02**	**0.02**	**0.34**	**0.02**	**0.02**	**0.36**	**0.03**	**0.02**	**0.17**
**TGP**				**0.05** +	**0.02**	**0.06**	**0.05** *	**0.02**	**0.04**	**0.05** *	**0.02**	**0.04**	**0.05** *	**0.02**	**0.04**
**FGA**				**0.04**	**0.02**	**0.14**	**0.03**	**0.02**	**0.21**	**0.03**	**0.02**	**0.19**	**0.03**	**0.02**	**0.16**
**TGP × FGA**							**0.03** *	**0.02**	**0.04**	**0.03** +	**0.02**	**0.05**	**0.03** *	**0.02**	**0.04**
**FGA × unemployment**										**0.03**	**0.02**	**0.23**	**0.03**	**0.02**	**0.16**
**TGP × unemployment**										**−0.01**	**0.02**	**0.65**	**−0.01**	**0.02**	**0.55**
**TGP × FGA × unemployment**													**−0.03** +	**0.02**	**0.06**
df	7, 167412.98			3, 56877.00			1, 36458.80			2, 20586.93			1, 20965.60		
*F*‐value	4.64 (*p* < 0.001)***			3.53 (*p* = 0.01)*			4.38 (*p* = 0.04)*			0.73 (*p* = 0.48)			3.45 (*p* =0.06)+		
R²	0.02			0.02			0.02			0.03			0.03		
Adjusted R²	0.01			0.02			0.02			0.02			0.02		

*** *p* < 0.001

** *p* < 0.01

* *p* < 0.05,

^+^ *p* < 0.10

**Table 6:** Multiple regression models: Effects on deviance between aspired and attained VET position (DV3_deviance).

Predictor	Model 1				Model 2				Model 3				Model 4				Model 5			
	*β*	*SE*	*OR*	*p*	*β*	*SE*	*OR*	*p*	*β*	*SE*	*OR*	*p*	*β*	*SE*	*OR*	*p*	*β*	*SE*	*OR*	*p*
Intercept	−0.36+	0.20	0.70	0.07	−0.37+	0.20	0.69	0.07	−0.37+	0.20	0.69	0.07	−0.37+	0.20	0.69	0.07	−0.37+	0.20	0.69	0.07
Leaving certificate	−0.19	0.15	0.83	0.21	−0.18	0.15	0.84	0.25	−0.18	0.15	0.84	0.25	−0.18	0.15	0.84	0.25	−0.18	0.15	0.84	0.25
Aspired type of VET	0.23	0.14	1.26	0.10	0.24	0.14	1.27	0.10	0.24	0.14	1.27	0.10	0.23	0.14	1.26	0.11	0.23	0.14	1.26	0.11
Wave	−0.93***	0.13	0.40	< 0.001	−0.92***	0.13	0.40	< 0.001	−0.92***	0.13	0.40	< 0.001	−0.92***	0.13	0.40	< 0.001	−0.91***	0.13	0.40	< 0.001
Migration background	0.11	0.14	1.12	0.44	0.10	0.14	1.11	0.47	0.10	0.14	1.11	0.46	0.10	0.14	1.11	0.47	0.11	0.14	1.11	0.47
Final grade	0.22***	0.05	1.25	< 0.001	0.23***	0.05	1.26	< 0.001	0.23***	0.05	1.26	< 0.001	0.23***	0.05	1.26	< 0.001	0.23***	0.05	1.26	< 0.001
SES	0.10+	0.05	1.10	0.07	0.10+	0.05	1.10	0.06	0.10+	0.05	1.10	0.06	0.10+	0.05	1.10	0.06	0.10+	0.05	1.10	0.06
Sex	−0.01	0.11	0.99	0.93	−0.01	0.11	0.99	0.95	−0.01	0.11	0.99	0.95	−0.01	0.11	0.99	0.94	−0.01	0.11	0.99	0.94
**Unemployment**					**0.03**	**0.05**	**1.03**	**0.59**	**0.03**	**0.05**	**1.03**	**0.59**	**0.03**	**0.05**	**1.03**	**0.60**	**0.03**	**0.05**	**1.03**	**0.57**
**TGP**					**0.08**	**0.06**	**1.10**	**0.16**	**0.08**	**0.06**	**1.08**	**0.17**	**0.08**	**0.06**	**1.08**	**0.17**	**0.08**	**0.06**	**1.08**	**0.17**
**FGA**					**−0.00**	**0.05**	**1.00**	**0.95**	**−0.00**	**0.05**	**1.00**	**0.95**	**−0.00**	**0.05**	**1.00**	**0.96**	**−0.00**	**0.05**	**1.00**	**0.97**
**TGP × FGA**									**−0.00**	**0.04**	**01.00**	**0.98**	**−0.00**	**0.04**	**1.00**	**0.94**	**−0.00**	**0.04**	**1.00**	**0.94**
**FGA × unemployment**													**0.01**	**0.06**	**1.01**	**0.86**	**0.01**	**0.06**	**1.01**	**0.83**
**TGP × unemployment**													**0.02**	**0.05**	**1.02**	**0.72**	**0.02**	**0.05**	**1.02**	**0.74**
**TGP × FGA × unemployment**																	**−0.01**	**0.04**	**0.99**	**0.81**
df	7, 47910.20				3, 13225.44				1, 2028.23				2, 8228.10				1, 3113.90			
*F*‐value	11.16*** (*p* < 0.001)				0.85 (*p* = 0.47)				0.00 (*p* = 0.98)				0.13 (*p* = 0.88)				0.06 (*p* = 0.81)			
Pseudo R²	0.04				0.04				0.04				0.04				0.04			
Adjusted Pseudo R²	0.03				0.03				0.03				0.03				0.03			

*Note:* ***p < 0.001

**p < 0.01

^+^ *p* < 0.10

**Table 7:** Multiple regression models: Effects on satisfaction with their VET position (DV4_satisfaction).

Predictor	Model 1			Model 2			Model 3			Model 4			Model 5		
	*β*	*SE*	*p*	*β*	*SE*	*p*	*β*	*SE*	*p*	*β*	*SE*	*p*	*β*	*SE*	*p*
Intercept	0.07	0.10	0.47	0.08	0.10	0.46	0.06	0.10	0.53	0.06	0.10	0.54	0.06	0.10	0.54
leaving certificate	0.05	0.08	0.51	0.06	0.08	0.48	0.06	0.08	0.45	0.06	0.08	0.44	0.06	0.08	0.44
Aspired type of VET	−0.03	0.07	0.68	−0.03	0.07	0.61	−0.04	0.07	0.57	−0.04	0.07	0.58	−0.04	0.07	0.58
Wave	−0.02	0.06	0.66	−0.03	0.06	0.62	−0.03	0.06	0.59	−0.03	0.06	0.59	−0.03	0.06	0.59
migration background	−0.17*	0.07	0.02	−0.19*	0.07	0.01	−0.19*	0.07	0.01	−0.19*	0.07	0.01	−0.19*	0.07	0.01
final grade	−0.03	0.02	0.30	−0.02	0.02	0.47	−0.02	0.02	0.44	−0.02	0.02	0.44	−0.02	0.02	0.44
SES	−0.03	0.03	0.19	−0.03	0.02	0.17	−0.03	0.02	0.20	−0.03	0.02	0.19	−0.03	0.02	0.19
Sex	−0.16**	0.05	0.002	−0.15**	0.05	0.004	−0.15**	0.05	0.004	−0.15**	0.05	0.004	−0.15**	0.05	0.004
**Unemployment**				**−0.02**	**0.02**	**0.38**	**−0.02**	**0.02**	**0.37**	**−0.02**	**0.02**	**0.37**	**−0.02**	**0.02**	**0.40**
**TGP**				**0.05** +	**0.03**	**0.07**	**0.05** *	**0.03**	**0.04**	**0.05** *	**0.03**	**0.04**	**0.05** *	**0.03**	**0.04**
**FGA**				**0.05** +	**0.03**	**0.07**	**0.04**	**0.03**	**0.11**	**0.04**	**0.03**	**0.10**	**0.04**	**0.03**	**0.10**
**TGP × FGA**							**0.04** *	**0.02**	**0.03**	**0.04** *	**0.02**	**0.03**	**0.04** *	**0.02**	**0.03**
**FGA × unemployment**										**0.02**	**0.03**	**0.51**	**0.02**	**0.03**	**0.50**
**TGP × unemployment**										**−0.02**	**0.03**	**0.47**	**−0.02**	**0.03**	**0.46**
**TGP × FGA × unemployment**													**−0.00**	**0.02**	**0.91**
df	7, 46036.56			3, 15159.07			1, 4527.46			2, 4525.37			1, 1803.94		
*F*‐value	2.42 (*p* = 0.02)*			3.51 (*p* = 0.01)*			4.63 (*p* = 0.03)*			0.34 (*p* = 0.71)			0.01 (*p *= 0.91)		
R²	0.01			0.02			0.02			0.02			0.02		
Adjusted R²	0.01			0.01			0.01			0.01			0.01		

***p < 0.001

** *p* < 0.01

* *p* < 0.05,

^+^ *p* < 0.10

We found effects of TGP on two indicators of VET success: TGP positively influences the self‐reported concordance between aspired and attained VET position (DV2_concordance *β* = 0.05, *p* = 0.04) as well as adolescents’ VET satisfaction (DV4_satisfaction; *β* = 0.05, *p* = 0.04). Effects on the start of a VET position (DV1_start; *β* = −0.01, *p* = 0.84) and on the objective deviance of the attained from the aspired VET position (DV3_deviance; *β* = 0.08, *p* = 0.17) are nonsignificant. These results provide partly evidence for *H1*, indicating that TGP is a helpful goal‐striving resource for some types of transition success. Evidence for *H2*, on the other hand, was not found in this study. FGA does not significantly influence either of the outcome variables examined.

Lastly, we detected interaction effects of TGP and FGA in the same models that revealed positive TGP effects. Both the interaction effects on DV2_concordance (*β* = 0.03, *p* = 0.04) as well as on DV4_satisfaction (*β* = 0.04, *p* = 0.03) are positive. Simple slope analyses revealed that TGP effects are only significant when FGA is average or above and that FGA effects become significant when TGP is above average (Long 2019; Nguyen 2020). These results provide evidence for *H3* and the CH, indicating that adolescents who have access to a wide range of goal‐striving strategies are more likely to succeed at the school‐to‐training transition.

#### 3.2.2 Moderation of Goal‐Striving Effects by Structural Obstacles

Next, we present how the effects of goal‐striving resources are moderated by unemployment. In *H4* and *H5* we examined two‐way interaction effects of each goal‐striving resource with unemployment. These effects are nonsignificant in all models, suggesting that neither TGP nor FGA influences on transition success differ between structural contexts.

**Figure 2:**
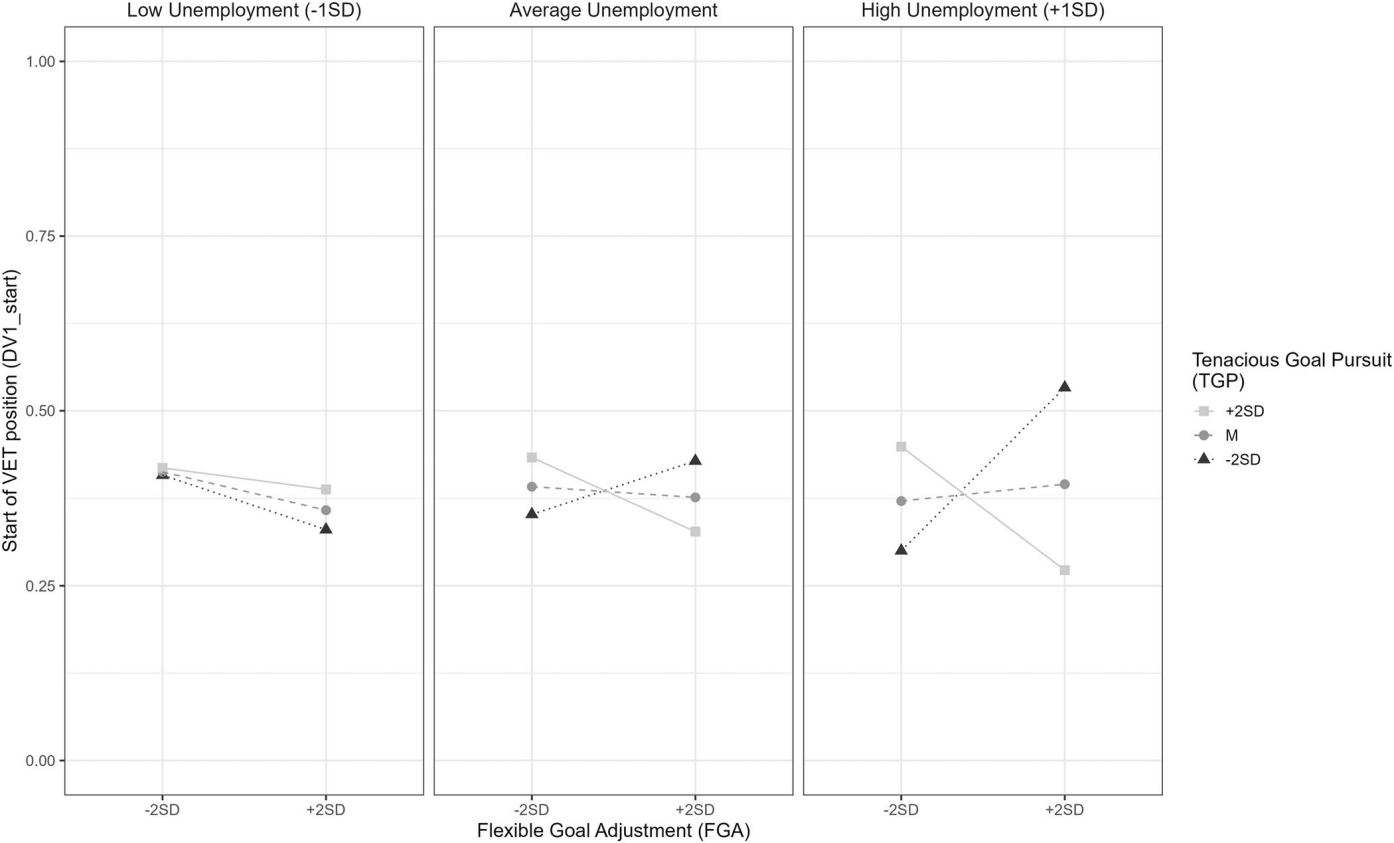
Three‐Way Interaction Effect TGP × FGA × Unemployment on DV1_start.

However, we did find some empirical evidence for *H6*, predicting a three‐way interaction between FGA, TGP and local unemployment. Firstly, we revealed a marginally significant negative TGP × FGA × unemployment interaction effect (*β* = −0.06, *p* = 0.097) on the chance of starting VET (DV1_start). Specifically, simple slope analysis revealed a negative TGP × FGA interaction if unemployment is high (+1SD). In such conditions, we found a positive effect of FGA on DV1_start only when TGP levels are low (−2SD; *β* = 0.25, *p* = 0.07) and a negative effect of TGP only when FGA is high (+1SD; *β* = −0.17, *p* = 0.06; Figure 2). In more favorable contexts, neither TGP nor FGA show a significant effect, regardless of the level of the other predictor. Hence, when unemployment is high, a combination of high FGA and low TGP seems to be most useful for the start of a VET position, whereas high scores in both are most detrimental. These findings are consistent with one of the predictions we made about three‐way interaction effects between our focal variables.

**Figure 3:**
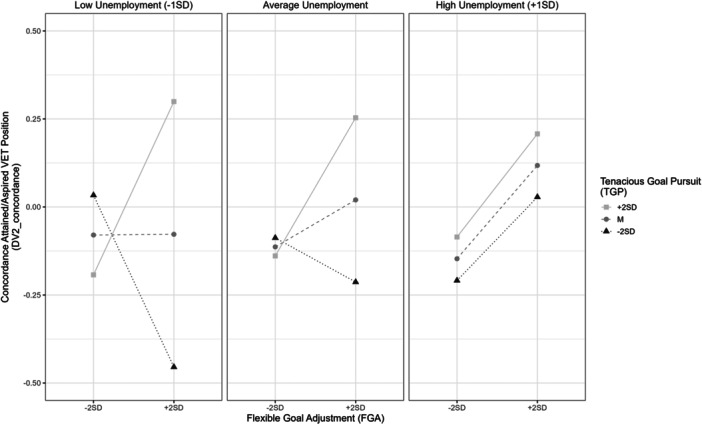
Three‐Way Interaction Effect TGP × FGA × Unemployment on DV2_concordance.

Apart from that, we found a negative TGP×FGA×unemployment interaction effect (*β* = −0.03, *p* = 0.06) on DV2_concordance. In this case, the positive TGP×FGA interaction, as described in the previous section, exists only when unemployment is low (−1SD) or average and vanishes when unemployment is high (+1SD). When unemployment is low (−1SD), very low TGP (−2SD) even leads to a negative FGP effect on DV2_concordance, hinting at the maladaptivity of FGA in favorable conditions. In contexts with high unemployment, FGA and TGP do not have an impact on transition success, regardless of the level of their counterpart, suggesting that in very unfavorable conditions goal‐striving resources of any type and in any combination lose usefulness. Although we expected the positive TGP×FGA interaction effect to be more—not less—pronounced in unfavorable conditions, these results do not contradict the general assumption that the effectiveness of goal‐striving resources depends on structural obstacles. We will discuss these relations further in the next section (Figure 3).

## 4 Discussion

Overall, our study showed that the two goal‐striving resources TGP and FGA aid the school‐to‐training‐transition. In line with the theoretical argument provided by Brandstädter and colleagues (Brandtstädter and Renner 1990; Brandtstädter and Rothermund 2002), their usefulness seems to depend on each other, on the degree of structural obstacles and on the success indicator under examination.

### 4.1 The Relevance of Goal‐Striving Resources for the School‐to‐Vet Transition

We found evidence for a main effect of TGP but not FGA on two indicators of transition success, the concordance between aspired and attained VET position (DV2_concordance) as well as VET satisfaction (DV4_satisfaction). In the same two models, we also revealed positive TGP × FGA interaction effects. These findings indicate that the influence of one goal‐striving resource depends on the other: The simultaneous availability of both TGP and FGA enables adolescents to choose the most helpful goal‐striving strategy (Bailly et al. 2016; Sahdra et al. 2022). If scores in one disposition are low, though, the other also loses usefulness. Simple slope analyses and the main effect pattern (Lorah 2020) reveal that this dependency is particularly pronounced for FGA, as flexibility is impactful only if TGP is above average. In any case, our study stresses the importance of taking a comprehensive view on goal‐striving: to be successful during their educational transition, adolescents need access to a range of strategies. Hence, both tenacious as well as flexible goal‐striving behaviors should be taught to students, so that they can succeed in their transition to training.

### 4.2 The Moderating Role of Structural Obstacles

The second goal of our study was to examine how the impact of goal‐striving resources on the school‐to‐VET transition is moderated by structural obstacles. Whereas we did not provide evidence for any two‐way interaction effects (H4 and H5), we found significant TGP × FGA × unemployment interactions on two indicators of VET success (H6): DV1_start and DV2_concordance. These findings indicate that the TGP × FGA interaction but not the effect of TGP or FGA themselves depends on structural obstacles in the local environment. These null‐results—although unexpected—are not surprising, as we showed in the previous section that TGP and FGA effects are dependent on each other.

We found two negative three‐way interaction effects. We draw three conclusions from the effect on DV2_concordance: Firstly, in favorable conditions, flexibility becomes detrimental when tenacity is not available to complement it. This finding is in line with the assumption that FGA is least helpful when goal attainment is highly likely. Secondly, on average conditions, it is best to have access to a wide range of strategies to react ideally to any circumstances. Thirdly, when structural obstacles are high, there might be little chance of goal achievement and hence FGA, TGP and their combination loses its usefulness. In addition to these insights, the three‐way interaction effect on DV1_start reveals another pattern: We found a negative TGP × FGA interaction, which only exists when structural obstacles are high. In those contexts, it is detrimental to be highly flexible and highly tenacious, whereas it is most adaptive to score high on FGA and low on TGP. When it is unlikely to start VET due to high local unemployment, high flexibility might be the only way to succeed. However, high additional tenacity would counteract its positive influence, as it works against the attempt to adapt one's goal to the prevailing structural obstacles. Instead, high FGA and TGP might result in a state of complete inactivity. We understand this mechanism as a more pronounced version of the one explained before. When the chances of obtaining a training place are low, high levels of both dispositions not only lose their advantage but become a disadvantage. In the next section we explore possible explanations for the differences observed between outcomes.

In any case, these three‐way interaction effects reveal a double disadvantage: Adolescents who live in vulnerable contexts cannot fall back on their individual resources to tackle these structural obstacles. At best, these resources might not have any impact, at worst they are even harmful in deprived contexts.

### 4.3 Differences Between Indicators of Transition Success

Our study revealed a strong dependency of effects on the outcome variable under examination, indicating that the susceptibility to goal‐striving strategies differs between transition goals. One distinction can be made between objective and subjective indicators of success: Whereas objective aspiration‐attainment‐concordance is not influenced by goal‐striving resources, self‐reported concordance is. Likewise, self‐reported VET satisfaction is significantly influenced by goal‐striving resources. These findings indicate that TGP and FGA might be especially relevant to adolescents’ *perception* of how well they handle the transition process and less important to actual goal attainment.

Nevertheless, we found a significant three‐way interaction effect on the objective start of VET but not on the other objective success indicator (DV3_deviance). We theorize that this is due to their differences in difficulty. In detrimental labor‐market conditions high flexibility might help attain any VET position but not the desired one. Hence, the more attainable an objective goal, the more likely it might be influenced by goal‐striving resources.

Lastly, it is interesting that the TGP×FGA interaction effect on DV4_satisfaction is not moderated by structural obstacles whereas that on DV2_concordance is. We believe that adolescents adjust their satisfaction to structural obstacles, meaning that under difficult circumstances they are satisfied with less as they take these challenges into account. Consequently, goal‐striving effects on satisfaction remains independent from structural obstacles.

Overall, these diverse findings stress the complexity of goal‐striving influences on transition success. Although we can only theorize about the mechanisms driving these differences, they underline the problem with one‐size‐fits‐all solutions when focusing on developmental tasks, as not every resource supports every type of success.

### 4.4 Limitations and Future Directions

Although our study sheds light on the interplay between goal‐striving resources and structural obstacles during the school‐to‐training transition, we must acknowledge its limitations. Our analyses are based on multiple imputation of missing information, which can introduce bias (van Buuren 2018; Woods et al. 2023). In robustness analyses we therefore showed that the alternative use of listwise‐deletion models does not substantially change the results (Appendix C).

Furthermore, effect sizes are considerably small (Cohen 1988; McFadden 1977). However, they increase when examining sublevels of moderator variables, indicating that effects depend on further individual or contextual conditions. Future research should put effort into identifying types of structural obstacles and individual resources that moderate the influence of goal‐striving strategies on successful school‐to‐VET transitions. For instance, in educational systems characterized by early stratification into standardized trajectories (e.g., Germany), goal‐striving resources might not be as useful as in more permeable transition systems (e.g., the UK), where educational pathways are more flexible and hence more susceptible to individual control (Schoon and Heckhausen 2019). Thus, the rigidity of the educational system might be another structural obstacle that moderates goal‐striving effects on transition success. Replicating our approach in different educational contexts would be a valuable avenue for further understanding the link between individual resources and structural constraints.

Apart from that, we were able to show that the effectiveness of goal‐striving resources depends on the developmental goal under examination. Further research could lead to a deeper understanding of the mechanisms behind these differences by systematically comparing effects on different transition outcomes, including subjective and objective ones as well as success indicators of varying difficulties.

Lastly, our results cannot speak to the causality of the effects. However, the use of a longitudinal study setup ensures their assumed directionality. With panel data such as the one we used this is a necessary compromise (Berrington 2006).

## 5 Conclusion

In summary, we were able to show that individual goal‐striving resources can be a source of support for adolescents transitioning to VET. However, we also revealed that their usefulness depends on the developmental goal, on local structural obstacles and on the interplay with other resources. Consequently, strengthening TGP or FGA will not be helpful to all adolescents and might even be harmful in certain conditions. More research in this area is necessary to understand these complex mechanisms and be able to offer targeted support to adolescents.

## Ethics Statement

The NEPS study is conducted under the supervision of the German Federal Commissioner for Data Protection and Freedom of Information (BfDI) and in coordination with the German Standing Conference of the Ministers of Education and Cultural Affairs (KMK) and—in the case of surveys at schools—the Educational Ministries of the respective Federal States. All data collection procedures, instruments and documents were checked by the data protection unit of the Leibniz Institute for Educational Trajectories (LIfBi). The necessary steps are taken to protect participants’ confidentiality according to national and international regulations of data security.

## Consent

Participation in the NEPS study is voluntary and based on the informed consent of participants. This consent to participate in the NEPS study can be revoked at any time.

## Conflicts of Interest

The authors declare no conflicts of interest.

## Supporting information


**Appendix A Long Scale Multiple Regression Models. Appendix B Multilevel Models. Appendix C Listwise Deletion Multiple Regression Models**.

## Data Availability

The individual data required to replicate the results of this study are available as a scientific use file from the Research Data Centre of the Leibniz Institute of Educational Trajectories (Starting Cohort Grade 5, https://doi.org/10.5157/NEPS:SC3:12.1.0, and Starting Cohort Grade 9, https://doi.org/10.5157/NEPS:SC4:13.0.0; see Blossfeld and Roßbach 2019). Additionally, the regional data is publicly available at the Federal Employment Agency (BA) and the Federal Institute for Research on Building, Urban Affairs and Spatial Development (BBSR). For more information, visit: https://www.neps-data.de/Mainpage, https://statistik.arbeitsagentur.de/DE/Navigation/Service/English-Site/English-Site-Nav.html and https://www.bbsr.bund.de/BBSR/EN/home/_node.html.

## Endnotes

1 This paper uses data from the National Educational Panel Study (NEPS): Starting Cohort Grade 5, https://doi.org/10.5157/NEPS:SC3:12.1.0, and Starting Cohort Grade 9, https://doi.org/10.5157/NEPS:SC4:13.0.0 (see Blossfeld and Roßbach 2019). From 2008 to 2013, NEPS data was collected as part of the Framework Program for the Promotion of Empirical Educational Research funded by the German Federal Ministry of Education and Research (BMBF). As of 2014, NEPS is carried out by the Leibniz Institute for Educational Trajectories (LIfBi) at the University of Bamberg in cooperation with a nation‐wide network.
