# Supplementary material for: Successful School‐to‐Training Transitions—Can Individual Goal‐Striving Resources Compensate for Structural Obstacles in the Local Context?
Source: J Adolesc. 2025 Aug 12;97(8):2246–63. doi: 10.1002/jad.70037 (PMC12682238; doi:10.1002/jad.70037)
Supplement: Supplementary file 1 — Appendix A Long Scale Multiple Regression Models. Appendix B Multilevel Models. Appendix C Listwise Deletion Multiple Regression Models. [file JAD-97-2246-s001.docx]

**Appendix A**

**Long Scale Multiple Regression Models**

**Table A1**

*Modelfit Indices of Short versus Long Scales*

| Fit Indices | 5-item-scales | 3-item-scales |
| --- | --- | --- |
| Χ² | 653.978 (p < .001) | 62.096 (p < .001) |
| RMSEA (robust) | 0.073 [0.068; 0.078] | 0.044 [0.034; 0.056] |
| SRMR | 0.048 | 0.022 |
| CFI (robust) | 0.882 | 0.974 |
| TLI | 1.000 | 1.000 |
| AIC | 83351.324 | 50599.731 |
| BIC | 83541.237 | 50716.129 |
| Cronbach α TGP | .69 | .55 |
| Cronbach α FGA | .67 | .57 |

*Note.* Results are based on Confirmatory Factor Analyses (CFA)

**Table A2**

*Multiple Regression Models: Effects on DV1_start (Long Scales)*

|  | Model 1 | | | Model 2 | | | Model 3 | | | Model 4 | | | Model 5 | | |
| --- | --- | --- | --- | --- | --- | --- | --- | --- | --- | --- | --- | --- | --- | --- | --- |
|  | β | *SE* | *p* | β | *SE* | *p* | β | *SE* | *p* | β | *SE* | *p* | β | *SE* | *p* |
| Intercept | −0.46** | .16 | .004 | −0.44** | .16 | .007 | −0.43** | .16 | .008 | −0.44** | .16 | .008 | −0.45** | .16 | .007 |
| Leaving certificate | 0.69*** | .13 | < .001 | 0.67*** | .13 | < .001 | 0.68*** | .13 | < .001 | 0.68*** | .13 | < .001 | 0.69*** | .13 | < .001 |
| Aspired type of VET | 1.87*** | .11 | < .001 | 1.86*** | .11 | < .001 | 1.87*** | .11 | < .001 | 1.87*** | .11 | < .001 | 1.87*** | .11 | < .001 |
| Wave | −0.24* | .11 | .04 | −0.25* | .11 | .04 | −0.24* | .11 | .04 | −0.24* | .11 | .04 | −0.25* | .11 | .03 |
| Migration background | −0.54*** | .12 | < .001 | −0.54*** | .12 | < .001 | −0.54*** | .12 | < .001 | −0.54*** | .12 | < .001 | −0.53*** | .12 | < .001 |
| final grade | −0.21*** | .05 | < .001 | −0.21*** | .05 | < .001 | −0.20*** | .05 | < .001 | −0.20*** | .05 | < .001 | −0.20*** | .05 | < .001 |
| SES | −0.08 | .05 | .14 | −0.08 | .05 | .14 | −0.08 | .05 | .15 | −0.08 | .05 | .14 | −0.08 | .05 | .16 |
| Sex | 0.02 | .10 | .85 | 0.02 | .11 | .87 | 0.02 | .11 | .87 | 0.02 | .11 | .88 | 0.02 | .11 | .88 |
| **Unemployment** |  |  |  | **−0.03** | **.05** | **.48** | **−0.03** | **.05** | **.49** | **−0.03** | **.05** | **.49** | **−0.00** | **.05** | **.98** |
| **TGP** |  |  |  | **0.02** | **.05** | **.65** | **0.02** | **.05** | **.70** | **0.02** | **.05** | **.66** | **0.03** | **.05** | **.61** |
| **FGA** |  |  |  | **−0.03** | **.06** | **.54** | **−0.03** | **.06** | **.58** | **−0.03** | **.06** | **.58** | **−0.03** | **.06** | **.65** |
| **TGP×FGA** |  |  |  |  |  |  | **−0.04** | **.04** | **.32** | **−0.04** | **.04** | **.35** | **−0.03** | **.04** | **.51** |
| **FGA×unemployment** |  |  |  |  |  |  |  |  |  | **0.02** | **.06** | **.70** | **0.03** | **.06** | **.60** |
| **TGP×unemployment** |  |  |  |  |  |  |  |  |  | **−0.07** | **.06** | **.23** | **−0.07** | **.06** | **.19** |
| **TGP×FGA×unemployment** |  |  |  |  |  |  |  |  |  |  |  |  | **−0.07^+^** | **.04** | **.05** |
| df | 7, 7499.82 | | | 3, 3923.73 | | | 1, 1423.88 | | | 2, 1306.20 | | | 1, 1140.32 | | |
| F-value | 57.00 (*p* < .001)*** | | | 0.32 (*p* = .81) | | | 1.01 (*p* = .32) | | | 0.72 (*p* = .49) | | | 3.77 (*p* = .05)^+^ | | |
| Pseudo R² | .16 | | | .16 | | | .16 | | | .16 | | | .16 | | |
| Adjusted Pseudo R² | .15 | | | .15 | | | .15 | | | .15 | | | .15 | | |

*** *p* < .001, ** *p* < .01, * *p* < .05, ^+^ *p* < .10

**Table A2**

*Multiple Regression Models: Effects on DV2_concordance (Long Scales)*

| Predictor | Model 1 | | | Model 2 | | | Model 3 | | | Model 4 | | | Model 5 | | |
| --- | --- | --- | --- | --- | --- | --- | --- | --- | --- | --- | --- | --- | --- | --- | --- |
|  | β | *SE* | *p* | β | *SE* | *p* | β | *SE* | *p* | β | *SE* | *p* | β | *SE* | *p* |
| Intercept | −0.01 | .10 | .91 | −0.03 | .10 | .76 | −0.04 | .10 | .70 | −0.04 | .10 | .70 | −0.04 | .10 | .68 |
| Leaving certificate | 0.16* | .08 | .04 | 0.18* | .08 | .03 | 0.18* | .08 | .03 | 0.18* | .08 | .02 | 0.18* | .08 | .02 |
| Aspired type of VET | −0.07 | .07 | .28 | −0.07 | .07 | .33 | −0.07 | .07 | .32 | −0.07 | .07 | .31 | −0.07 | .07 | .33 |
| Wave | 0.08 | .05 | .13 | 0.09 | .05 | .11 | 0.09 | .05 | .11 | 0.09 | .05 | .11 | 0.09 | .05 | .10 |
| Migration background | −0.25*** | .07 | < .001 | −0.26*** | .07 | < .001 | −0.26*** | .07 | < .001 | −0.26*** | .07 | < .001 | −0.26*** | .07 | < .001 |
| Final grade | −0.03 | .02 | .23 | −0.02 | .02 | .36 | −0.02 | .02 | .34 | −0.02 | .02 | .35 | −0.02 | .02 | .34 |
| SES | −0.05^+^ | .03 | .06 | −0.05^+^ | .03 | .06 | −0.05^+^ | .03 | .07 | −0.05^+^ | .03 | .07 | −0.05^+^ | .03 | .07 |
| Sex | −0.13* | .05 | .01 | −0.12* | .05 | .02 | −0.12* | .05 | .02 | −0.12* | .05 | .02 | −0.12* | .05 | .02 |
| **Unemployment** |  |  |  | **0.02** | **.02** | **.35** | **0.02** | **.02** | **.35** | **0.02** | **.02** | **.37** | **0.03** | **.02** | **.22** |
| **TGP** |  |  |  | **0.04^+^** | **.03** | **.08** | **0.05^+^** | **.03** | **.07** | **0.05^+^** | **.03** | **.07** | **0.05^+^** | **.03** | **.06** |
| **FGA** |  |  |  | **0.05*** | **.02** | **.04** | **0.05*** | **.02** | **.06** | **0.05^+^** | **.02** | **.05** | **0.05*** | **.02** | **.04** |
| **TGP×FGA** |  |  |  |  |  |  | **0.02** | **.02** | **.17** | **0.02** | **.02** | **.21** | **0.02** | **.02** | **.20** |
| **FGA×unemployment** |  |  |  |  |  |  |  |  |  | **0.02** | **.02** | **.41** | **0.02** | **.02** | **.33** |
| **TGP×unemployment** |  |  |  |  |  |  |  |  |  | **−0.01** | **.03** | **.84** | **−0.01** | **.03** | **.77** |
| **TGP×FGA×unemployment** |  |  |  |  |  |  |  |  |  |  |  |  | **−0.02** | **.02** | **.19** |
| df | 7, 172990.06 | | | 3, 47576.19 | | | 1, 19735.36 | | | 2, 19202.75 | | | 1, 16131.47 | | |
| F-value | 4.67 (p < .001)*** | | | 4.54 (p = .003)* | | | 1.85 (p = .17) | | | 0.35 (p = .70) ^+^ | | | 1.71 (p = .19) | | |
| R² | .02 | | | .02 | | | .03 | | | .03 | | | .03 | | |
| Adjusted R² | .01 | | | .02 | | | .02 | | | .02 | | | .02 | | |

*** *p* < .001, ** *p* < .01, * *p* < .05, ^+^ *p* < .10

**Table A3**

*Multiple Regression Models: Effects on DV3_deviance (Long Scales)*

| Predictor | Model 1 | | | Model 2 | | | Model 3 | | | Model 4 | | | Model 5 | | |
| --- | --- | --- | --- | --- | --- | --- | --- | --- | --- | --- | --- | --- | --- | --- | --- |
|  | β | *SE* | *p* | β | *SE* | *p* | β | *SE* | *p* | β | *SE* | *p* | β | *SE* | *p* |
| Intercept | −0.37 | .20 | .07 | −0.38 | .21 | .07 | −0.39 | .21 | .06 | −0.39 | .21 | .06 | −0.39 | .21 | .06 |
| Leaving certificate | −0.18 | .15 | .24 | −0.17 | .15 | .26 | −0.17 | .15 | .27 | −0.17 | .16 | .27 | −0.17 | .16 | .27 |
| Aspired type of VET | 0.24^+^ | .14 | .097 | 0.23 | .14 | .11 | 0.23 | .14 | .11 | 0.23 | .14 | .11 | 0.23 | .14 | .11 |
| Wave | −0.92*** | .13 | < .001 | −0.91*** | .13 | < .001 | −0.91*** | .13 | < .001 | −0.91*** | .13 | < .001 | −0.91*** | .13 | < .001 |
| Migration background | 0.11 | .14 | .41 | 0.11 | .14 | .44 | 0.11 | .14 | .43 | 0.11 | .14 | .42 | 0.11 | .14 | .42 |
| Final grade | 0.22*** | .05 | < .001 | 0.24*** | .05 | < .001 | 0.24*** | .05 | < .001 | 0.24*** | .05 | < .001 | 0.24*** | .05 | < .001 |
| SES | 0.10^+^ | .05 | .05 | 0.11^+^ | .05 | .05 | 0.11^+^ | .05 | .05 | 0.11^+^ | .05 | .05 | 0.11^+^ | .05 | .05 |
| Sex | −0.02 | .11 | .87 | −0.01 | .11 | .90 | −0.01 | .11 | .91 | −0.01 | .11 | .90 | −0.01 | .11 | .90 |
| **Unemployment** |  |  |  | **0.02** | **.05** | **.66** | **0.02** | **.05** | **.66** | **0.02** | **.05** | **.68** | **0.02** | **.05** | **.65** |
| **TGP** |  |  |  | **0.10^+^** | **.06** | **.06** | **0.11^+^** | **.06** | **.05** | **0.11^+^** | **.06** | **.06** | **0.11^+^** | **.06** | **.06** |
| **FGA** |  |  |  | **−0.02** | **.06** | **.71** | **−0.02** | **.06** | **.66** | **−0.02** | **.06** | **.67** | **−0.02** | **.06** | **.68** |
| **TGP×FGA** |  |  |  |  |  |  | **0.02** | **.04** | **.59** | **0.02** | **.04** | **.63** | **0.02** | **.04** | **.63** |
| **FGA×unemployment** |  |  |  |  |  |  |  |  |  | **0.01** | **.06** | **.88** | **0.01** | **.06** | **.86** |
| **TGP×unemployment** |  |  |  |  |  |  |  |  |  | **0.01** | **.06** | **.85** | **0.01** | **.06** | **.87** |
| **TGP×FGA×unemployment** |  |  |  |  |  |  |  |  |  |  |  |  | **−0.01** | **.04** | **.87** |
| df | 7, 44849.90 | | | 3, 12147.24 | | | 1, 2990.94 | | | 2, 4833.64 | | | 1, 4005.19 | | |
| F-value | 11.21 (*p* < .001)*** | | | 1.31 (*p* = .27) | | | 0.28 (*p* = .59) | | | 0.05 (*p* = .95) | | | 0.03 (*p* = .87) | | |
| R² | .04 | | | .04 | | | .04 | | | .04 | | | .04 | | |
| Adjusted R² | .03 | | | .03 | | | .03 | | | .03 | | | .03 | | |

*** *p* < .001, ** *p* < .01, * *p* < .05, ^+^ *p* < .10

**Table A4**

*Multiple Regression Models: Effects on DV4_satisfaction (Long Scales)*

| Predictor | Model 1 | |  | Model 2 | |  | Model 3 | |  | Model 4 | |  | Model 5 | |  |
| --- | --- | --- | --- | --- | --- | --- | --- | --- | --- | --- | --- | --- | --- | --- | --- |
|  | *β* | *SE* | *p* | *β* | *SE* | *p* | *β* | *SE* | *p* | *β* | *SE* | *p* | *β* | *SE* | *p* |
| Intercept | 0.07 | .10 | .49 | 0.07 | .10 | .49 | 0.05 | .10 | .59 | 0.05 | .10 | .60 | 0.05 | .10 | .60 |
| Leaving certificate | 0.06 | .08 | .46 | 0.06 | .08 | .42 | 0.06 | .08 | .41 | 0.06 | .08 | .41 | 0.06 | .08 | .41 |
| Aspired type of VET | −0.03 | .07 | .67 | −0.03 | .07 | .62 | −0.04 | .07 | .58 | −0.04 | .07 | .60 | −0.04 | .07 | .59 |
| Wave | −0.03 | .06 | .64 | −0.03 | .06 | .58 | −0.03 | .06 | .57 | −0.03 | .06 | .56 | −0.03 | .06 | .56 |
| Migration background | −0.16* | .07 | .03 | −0.19* | .07 | .01 | −0.18* | .07 | .01 | −0.18* | .07 | .01 | −0.18* | .07 | .01 |
| Final grade | −0.03 | .02 | .27 | −0.02 | .02 | .45 | −0.02 | .02 | .42 | −0.02 | .02 | .42 | −0.02 | .02 | .42 |
| SES | −0.03 | .03 | .26 | −0.03 | .03 | .23 | −0.03 | .03 | .25 | −0.04 | .03 | .25 | −0.03 | .03 | .25 |
| Sex | −0.16** | .05 | .003 | −0.14** | .05 | .006 | −0.14** | .05 | .007** | −0.14** | .05 | .007 | −0.14** | .05 | .01 |
| **Unemployment** |  |  |  | **−0.02** | **.02** | **.29** | **−0.02** | **.02** | **.30** | **−0.02** | **.02** | **.31** | **−0.02** | **.02** | **.31** |
| **TGP** |  |  |  | **0.05^+^** | **.03** | **.08** | **0.05^+^** | **.03** | **.05** | **0.05^+^** | **.03** | **.05** | **0.05^+^** | **.03** | **.06** |
| **FGA** |  |  |  | **0.07**** | **.03** | **.01** | **0.07*** | **.03** | **.01** | **0.07*** | **.03** | **.01** | **0.07*** | **.03** | **.01** |
| **TGP×FGA** |  |  |  |  |  |  | **0.04*** | **.02** | **.04** | **0.04^+^** | **.02** | **.05** | **0.04^+^** | **.02** | **.05** |
| **FGA×unemployment** |  |  |  |  |  |  |  |  |  | **−0.00** | **.03** | **.98** | **−0.00** | **.03** | **.96** |
| **TGP×unemployment** |  |  |  |  |  |  |  |  |  | **−0.01** | **.03** | **.82** | **−0.01** | **.03** | **.83** |
| **TGP×FGA×unemployment** |  |  |  |  |  |  |  |  |  |  |  |  | **0.00** | **.02** | **.89** |
| df | 7, 34861.58 | | | 3, 14396.14 | | | 1, 2294.50 | | | 2, 4852.66 | | | 1, 946.55 | | |
| F-value | 2.31 (*p* = .02)* | | | 5.67 (*p* < .001)*** | | | 4.06 (*p* = .04)* | | | 0.04 (*p* = .96) | | | 0.02 (*p* = .89) | | |
| R² | .01 | | | .02 | | | .02 | | | .02 | | | .02 | | |
| Adjusted R² | .01 | | | .02 | | | .02 | | | .02 | | | .02 | | |

*** *p* < .001, ** *p* < .01, * *p* < .05, ^+^ *p* < .10

**Appendix B**

**Multilevel Models**

**Table B1**

*Generalized Linear Mixed Models: Effects on DV1_start*

| Predictor | Model 1 | | | Model 2 | | | Model 3 | | | Model 4 | | | Model 5 | | |
| --- | --- | --- | --- | --- | --- | --- | --- | --- | --- | --- | --- | --- | --- | --- | --- |
|  | *β* | *SE* | *p* | *β* | *SE* | *p* | *β* | *SE* | *p* | *β* | *SE* | *p* | *β* | *SE* | *p* |
| Intercept | −0.48** | .11 | .01 | −0.48** | .11 | .006 | −0.46** | .11 | .007 | −0.46** | .11 | .007 | −0.47** | .11 | .006 |
| Leaving certificate | 0.69*** | .15 | < .001 | 0.69*** | .15 | < .001 | 0.69*** | .15 | < .001 | 0.69*** | .15 | < .001 | 0.70*** | .15 | < .001 |
| Aspired type of VET | 1.96*** | .12 | < .001 | 1.96*** | .12 | < .001 | 1.97*** | .12 | < .001 | 1.97*** | .12 | < .001 | 1.97*** | .12 | < .001 |
| Wave | −0.27* | .14 | .046 | −0.27* | .14 | .04 | −0.27* | .14 | .049 | −0.27^+^ | .14 | .05 | −0.27* | .14 | .04 |
| Migration background | −0.52*** | .14 | < .001 | −0.52*** | .14 | < .001 | −0.52*** | .14 | < .001 | −0.52*** | .14 | < .001 | −0.51*** | .14 | < .001 |
| Final grade | −0.22*** | .06 | < .001 | −0.22*** | .06 | < .001 | −0.21*** | .06 | < .001 | −0.21*** | .06 | < .001 | −0.21*** | .06 | < .001 |
| SES | −0.08 | .06 | .16 | −0.08 | .06 | .16 | −0.08 | .06 | .16 | −0.08 | .06 | .16 | −0.08 | .06 | .16 |
| Sex | 0.03 | .12 | .82 | 0.02 | .12 | .83 | 0.02 | .12 | .84 | 0.02 | .12 | .85 | 0.02 | .12 | .85 |
| **Unemployment** |  |  |  | **−0.02** | **.06** | **.74** | **−0.02** | **.06** | **.75** | **−0.02** | **.06** | **.73** | **−0.01** | **.06** | **.91** |
| **TGP** |  |  |  | **0.00** | **.06** | **1.00** | **−0.01** | **.06** | **.90** | **−0.01** | **.06** | **.93** | **−0.00** | **.06** | **.96** |
| **FGA** |  |  |  | **−0.03** | **.06** | **.61** | **−0.02** | **.06** | **.66** | **−0.03** | **.06** | **.64** | **−0.02** | **.06** | **.69** |
| **TGP×FGA** |  |  |  |  |  |  | **−0.06** | **.04** | **.17** | **−0.06** | **.04** | **.17** | **−0.05** | **.04** | **.23** |
| **FGA×unemployment** |  |  |  |  |  |  |  |  |  | **0.04** | **.05** | **.52** | **0.04** | **.06** | **.46** |
| **TGP×unemployment** |  |  |  |  |  |  |  |  |  | **−0.04** | **.05** | **.44** | **−0.05** | **.05** | **.36** |
| **TGP× FGA×unemployment** |  |  |  |  |  |  |  |  |  |  |  |  | **−0.06^+^** | **.04** | **.097** |

*Note.* These analyses were conducted with STATA (StataCorp, 2021). Individuals (L1) were clustered within schools (L2). We used cluster-robust standard errors in all models. *** *p* < .001, ** *p* < .01, * *p* < .05, ^+^ *p* < .10

**Table B2**

*Linear Mixed Models: Effects on DV2_concordance*

| Predictor | Model 1 | | | Model 2 | | | Model 3 | | | Model 4 | | | Model 5 | | |
| --- | --- | --- | --- | --- | --- | --- | --- | --- | --- | --- | --- | --- | --- | --- | --- |
|  | *β* | *SE* | *p* | *β* | *SE* | *p* | *β* | *SE* | *p* | *β* | *SE* | *p* | *β* | *SE* | *p* |
| Intercept | −0.02 | .10 | .84 | −0.04 | .10 | .69 | −0.05 | .10 | .61 | −0.05 | .10 | .84 | −0.06 | .10 | .58 |
| Leaving certificate | 0.17* | .08 | .03 | 0.18* | .08 | .02 | 0.19* | .08 | .02 | 0.19* | .08 | .02 | 0.19* | .08 | .02 |
| Aspired type of VET | −0.07 | .07 | .29 | −0.07 | .07 | .32 | −0.07 | .07 | .30 | −0.07 | .07 | .29 | −0.07 | .07 | .31 |
| Wave | 0.09 | .05 | .10 | 0.09^+^ | .05 | .07 | 0.09^+^ | .05 | .08 | 0.09^+^ | .05 | .08 | 0.09^+^ | .05 | .08 |
| Migration background | −0.25*** | .07 | < .001 | −0.26*** | .07 | < .001 | −0.26*** | .07 | < .001 | −0.26*** | .07 | < .001 | −0.25*** | .07 | < .001 |
| Final grade | −0.03 | .03 | .27 | −0.02 | .03 | .40 | −0.02 | .03 | .38 | −0.02 | .03 | .38 | −0.02 | .03 | .36 |
| SES | −0.05^+^ | .03 | .07 | −0.04^+^ | .03 | .08 | −0.04^+^ | .03 | .09 | −0.04^+^ | .03 | .08 | −0.04^+^ | .03 | .09 |
| Sex | −0.12* | .05 | .02 | −0.12* | .05 | .02 | −0.12* | .05 | .02 | −0.12* | .05 | .02 | −0.12* | .05 | .02 |
| **Unemployment** |  |  |  | **0.03** | **.02** | **.30** | **0.03** | **.02** | **.31** | **0.02** | **.02** | **.32** | **0.04** | **.02** | **.15** |
| **TGP** |  |  |  | **0.05^+^** | **.02** | **.05** | **0.05*** | **.02** | **.04** | **0.05*** | **.02** | **.04** | **0.05*** | **.02** | **.03** |
| **FGA** |  |  |  | **0.04** | **.02** | **.16** | **0.03** | **.02** | **.23** | **0.03** | **.02** | **.21** | **0.03** | **.02** | **.17** |
| **TGP×FGA** |  |  |  |  |  |  | **0.03*** | **.02** | **.03** | **0.03*** | **.02** | **.04** | **0.03*** | **.02** | **.03** |
| **FGA×unemployment** |  |  |  |  |  |  |  |  |  | **0.03** | **.02** | **.20** | **0.03** | **.02** | **.14** |
| **TGP×unemployment** |  |  |  |  |  |  |  |  |  | **−0.01** | **.02** | **.66** | **−0.01** | **.02** | **.55** |
| **TGP×FGA×unemployment** |  |  |  |  |  |  |  |  |  |  |  |  | **−0.03*** | **.01** | **.04** |

*Note.* Individuals (L1) were clustered within schools (L2). We used cluster-robust standard errors in all models. *** *p* < .001, ** *p* < .01, * *p* < .05, ^+^ *p* < .10

**Table B3**

*Generalized Linear Mixed Models: Effects on DV3_deviance*

| Predictor | Model 1 | | | Model 2 | | | Model 3 | | | Model 4 | | | Model 5 | | |
| --- | --- | --- | --- | --- | --- | --- | --- | --- | --- | --- | --- | --- | --- | --- | --- |
|  | *β* | *SE* | *p* | *β* | *SE* | *p* | *β* | *p* | *SE* | *β* | *SE* | *p* | *β* | *SE* | *p* |
| Intercept | −0.36^+^ | .20 | .08 | −0.37^+^ | .21 | .07 |  |  |  | −0.37^+^ | .21 | .07 | −0.37^+^ | .21 | .07 |
| Leaving certificate | −0.19 | .16 | .21 | −0.18 | .16 | .25 |  |  |  | −0.18 | .16 | .25 | −0.18 | .16 | .25 |
| Aspired type of VET | 0.24^+^ | .14 | .09 | 0.24^+^ | .14 | .09 |  |  |  | 0.23^+^ | .14 | .096 | 0.23^+^ | .14 | .096 |
| Wave | −0.93*** | .14 | < .001 | −0.92*** | .14 | < .001 |  |  |  | −0.92*** | .14 | < .001 | −0.92*** | .14 | < .001 |
| Migration background | 0.11 | .15 | .47 | 0.10 | .15 | .50 |  |  |  | 0.10 | .15 | .49 | 0.10 | .15 | .49 |
| Final grade | 0.22*** | .05 | < .001 | 0.23*** | .05 | < .001 |  |  |  | 0.23*** | .05 | < .001 | 0.23*** | .05 | < .001 |
| SES | 0.10^+^ | .10 | .08 | 0.10 ^+^ | .10 | .08 |  |  |  | 0.10^+^ | .10 | .08 | 0.10^+^ | .10 | .08 |
| Sex | −0.01 | .11 | .93 | −0.01 | .11 | .96 |  |  |  | −0.01 | .11 | .94 | −0.01 | .11 | .95 |
| **Unemployment** |  |  |  | **0.03** | **.05** | **.60** |  |  |  | **0.02** | **.05** | **.62** | **0.03** | **.05** | **.58** |
| **TGP** |  |  |  | **0.08** | **.05** | **.17** |  |  |  | **0.08** | **.06** | **.17** | **0.08** | **.06** | **.17** |
| **FGA** |  |  |  | **−0.002** | **.06** | **.96** |  |  |  | **−0.002** | **.06** | **.97** | **−0.00** | **.06** | **.98** |
| **TGP×FGA** |  |  |  |  |  |  |  |  |  | **−0.002** | **.04** | **.95** | **−0.00** | **.04** | **.95** |
| **FGA×unemployment** |  |  |  |  |  |  |  |  |  | **0.01** | **.06** | **.86** | **0.01** | **.06** | **.84** |
| **TGP×unemployment** |  |  |  |  |  |  |  |  |  | **0.02** | **.05** | **.71** | **0.02** | **.05** | **.73** |
| **TGP×FGA×unemployment** |  |  |  |  |  |  |  |  |  |  |  |  | **−0.01** | **.04** | **.81** |

*Note.* These analyses were conducted with STATA (StataCorp, 2021). Individuals (L1) were clustered within schools (L2). We used cluster-robust standard errors in all models. Model 3 did not converge. *** *p* < .001, ** *p* < .01, * *p* < .05, ^+^ *p* < .10

**Table B4**

*Linear Mixed Models: Effects on DV4_satisfaction*

| Predictor | Model 1 | | | Model 2 | | | Model 3 | | | Model 4 | | | Model 5 | | |
| --- | --- | --- | --- | --- | --- | --- | --- | --- | --- | --- | --- | --- | --- | --- | --- |
|  | *β* | *SE* | *p* | *β* | *SE* | *p* | *β* | *SE* | *p* | *β* | *SE* | *p* | *β* | *SE* | *p* |
| Intercept | 0.07 | .10 | .48 | 0.07 | .10 | .46 | 0.06 | .10 | .55 | 0.06 | .10 | .56 | 0.06 | .10 | .56 |
| Leaving certificate | 0.05 | .08 | .49 | 0.06 | .08 | .46 | 0.06 | .08 | .43 | 0.06 | .08 | .42 | 0.06 | .08 | .42 |
| Aspired type of VET | −0.03 | .07 | .66 | −0.04 | .07 | .59 | −0.04 | .07 | .55 | −0.04 | .07 | .56 | −0.04 | .07 | .56 |
| Wave | −0.02 | .05 | .67 | −0.03 | .05 | .63 | −0.03 | .05 | .60 | −0.03 | .05 | .59 | −0.03 | .05 | .59 |
| Migration background | −0.17* | .07 | .02 | −0.19* | .07 | .01 | −0.19* | .07 | .01 | −0.19* | .07 | .01 | −0.19* | .07 | .01 |
| Final grade | −0.03 | .02 | .28 | −0.02 | .02 | .45 | −0.02 | .02 | .42 | −0.02 | .02 | .42 | −0.02 | .02 | .43 |
| SES | −0.03 | .02 | .18 | −0.03 | .02 | .16 | −0.03 | .02 | .19 | −0.03 | .02 | .18 | −0.03 | .02 | .18 |
| Sex | −0.15** | .05 | .003 | −0.15** | .05 | .004 | −0.15** | .05 | .004 | −0.15** | .05 | .004 | −0.15** | .05 | .004 |
| **Unemployment** |  |  |  | **−0.02** | **.02** | **.40** | **−0.02** | **.02** | **.39** | **−0.02** | **.02** | **.39** | **−0.02** | **.02** | **.43** |
| **TGP** |  |  |  | **0.05^+^** | **.03** | **.08** | **0.05^+^** | **.03** | **.05** | **0.05^+^** | **.03** | **.05** | **0.05^+^** | **.03** | **.05** |
| **FGA** |  |  |  | **0.05^+^** | **.03** | **.06** | **0.04** | **.03** | **.10** | **0.04^+^** | **.03** | **.09** | **0.04^+^** | **.03** | **.09** |
| **TGP×FGA** |  |  |  |  |  |  | **0.04*** | **.02** | **.02** | **0.04*** | **.02** | **.02** | **0.04*** | **.02** | **.02** |
| **FGA×unemployment** |  |  |  |  |  |  |  |  |  | **0.02** | **.03** | **.53** | **0.02** | **.03** | **.52** |
| **TGP×unemployment** |  |  |  |  |  |  |  |  |  | **−0.02** | **.03** | **.49** | **−0.02** | **.03** | **.49** |
| **TGP×FGA×unemployment** |  |  |  |  |  |  |  |  |  |  |  |  | **−0.00** | **.02** | **.90** |

*Note.* Individuals (L1) were clustered within schools (L2). We used cluster-robust standard errors in all models. *** *p* < .001, ** *p* < .01, * *p* < .05, ^+^ *p* < .10

**Appendix C**

**Listwise Deletion Multiple Regression Models**

**Table C1**

*Multiple Regression Models after Listwise Deletion: Effects on DV1_start*

| Predictor | Model 1 | | | Model 2 | | | Model 3 | | | Model 4 | | | Model 5 | | |
| --- | --- | --- | --- | --- | --- | --- | --- | --- | --- | --- | --- | --- | --- | --- | --- |
|  | *β* | *SE* | *p* | *β* | *SE* | *p* | *β* | *SE* | *p* | *β* | *SE* | *p* | *β* | *SE* | *p* |
| Intercept | −0.37 | .23 | .10 | −0.32 | .23 | .17 | −0.29 | .23 | .21 | −0.29 | .23 | .21 | −0.30 | .23 | .20 |
| Leaving certificate | 0.55* | .20 | .01 | 0.53* | .20 | .01 | 0.52* | .20 | .01 | 0.53* | .20 | .01 | 0.53* | .20 | .01 |
| Aspired type of VET | 2.05*** | .14 | < .001 | 2.03*** | .14 | < .001 | 2.03*** | .14 | < .001 | 2.03*** | .14 | < .001 | 2.04*** | .14 | < .001 |
| Wave | −0.32^+^ | .16 | .05 | −0.36* | .16 | .03 | −0.36* | .16 | .03 | −0.36* | .16 | .03 | −0.36* | .16 | .03 |
| Migration background | −0.79*** | .16 | < .001 | −0.80*** | .16 | < .001 | −0.80*** | .16 | < .001 | −0.80*** | .16 | < .001 | −0.79*** | .16 | < .001 |
| Final grade | −0.31*** | .06 | < .001 | −0.31*** | .06 | < .001 | −0.31*** | .06 | < .001 | −0.31*** | .06 | < .001 | −0.31*** | .06 | < .001 |
| SES | −0.09 | .06 | .15 | −0.10 | .06 | .12 | −0.10 | .06 | .11 | −0.10 | .06 | .11 | −0.10 | .06 | .13 |
| Sex | 0.07 | .14 | .62 | 0.06 | .14 | .68 | 0.05 | .14 | .71 | 0.05 | .14 | .74 | 0.05 | .14 | .74 |
| **Unemployment** |  |  |  | **−0.11^+^** | **.06** | **.06** | **−0.11^+^** | **.06** | **.07** | **−0.11^+^** | **.06** | **.06^+^** | **−0.07** | **.07** | **.30** |
| **TGP** |  |  |  | **−0.06** | **.07** | **.36** | **−0.07** | **.07** | **.32** | **−0.06** | **.07** | **.34** | **−0.06** | **.07** | **.34** |
| **FGA** |  |  |  | **0.03** | **.07** | **.67** | **0.03** | **.07** | **.61** | **0.03** | **.07** | **.63** | **0.04** | **.07** | **.55** |
| **TGP×FGA** |  |  |  |  |  |  | **−0.06** | **.05** | **.20** | **−0.06** | **.05** | **.19** | **−0.05** | **.05** | **.32** |
| **FGA×unemployment** |  |  |  |  |  |  |  |  |  | **0.05** | **.07** | **.41** | **0.07** | **.07** | **.30** |
| **TGP×unemployment** |  |  |  |  |  |  |  |  |  | **−0.05** | **.06** | **.45** | **−0.05** | **.07** | **.42** |
| **TGP×FGA×unemployment** |  |  |  |  |  |  |  |  |  |  |  |  | **−0.11*** | **.05** | **.04** |
| df | 7, 1921 | | | 3, 1918 | | | 1, 1917 | | | 2, 1915 | | | 1, 1914 | | |
| F-value | 44.23 (*p* < .001)*** | | | 1.48 (*p* = .22) | | | 1.64 (*p* = .20) | | | 0.44 (*p* = .65) | | | 4.31 (*p* = .04)* | | |
| Pseudo R² | .18 | | | .18 | | | .18 | | | .18 | | | .19 | | |
| Adjusted Pseudo R² | .17 | | | .17 | | | .17 | | | .17 | | | .17 | | |

*** *p* < .001, ** *p* < .01, * *p* < .05, ^+^ *p* < .10

**Table C2**

*Multiple Regression Models after Listwise Deletion: Effects on DV2_concordance*

| Predictor | Model 1 | | | Model 2 | | | Model 3 | | | Model 4 | | | Model 5 | | |
| --- | --- | --- | --- | --- | --- | --- | --- | --- | --- | --- | --- | --- | --- | --- | --- |
|  | *β* | *SE* | *p* | *β* | *SE* | *p* | *β* | *SE* | *p* | *β* | *SE* | *p* | *β* | *SE* | *p* |
| Intercept | −0.07 | .13 | .62 | −0.09 | .13 | .52 | −0.09 | .13 | .48 | −0.10 | .13 | .45 | −0.11 | .13 | .43 |
| Leaving certificate | 0.16 | .10 | .11 | 0.18^+^ | .10 | .08 | 0.18 ^+^ | .10 | .08 | 0.19^+^ | .10 | .07 | 0.19^+^ | .10 | .07 |
| Aspired type of VET | −0.02 | .08 | .77 | −0.02 | .08 | .81 | −0.02 | .08 | .79 | −0.02 | .08 | .82 | −0.01 | .08 | .87 |
| Wave | 0.16* | .07 | .01 | 0.17* | .07 | .01 | 0.17* | .07 | .01 | 0.17* | .07 | .01 | 0.17* | .07 | .01 |
| Migration background | −0.23** | .08 | .004 | −0.24** | .08 | .002 | −0.24** | .08 | .002 | −0.24** | .08 | .002 | −0.23** | .08 | .003 |
| Final grade | −0.04 | .03 | .15 | −0.03 | .03 | .24 | −0.03 | .03 | .23 | −0.03 | .03 | .22 | −0.03 | .03 | .23 |
| SES | −0.09** | .03 | .001 | −0.09** | .03 | .001 | −0.09** | .03 | .001 | −0.09** | .03 | .001 | −0.09** | .03 | .001 |
| Sex | −0.14* | .06 | .01 | −0.13* | .06 | .02 | −0.13* | .06 | .02 | −0.14* | .06 | .02 | −0.14* | .06 | .02 |
| **Unemployment** |  |  |  | **0.01** | **.03** | **.61** | **0.01** | **.03** | **.60** | **0.01** | **.03** | **.63** | **0.03** | **.03** | **.33** |
| **TGP** |  |  |  | **0.04** | **.03** | **.14** | **0.04** | **.03** | **.12** | **0.04** | **.03** | **.13** | **0.05** | **.03** | **.11** |
| **FGA** |  |  |  | **0.04** | **.03** | **.15** | **0.04** | **.03** | **.19** | **0.04** | **.03** | **.16** | **0.04** | **.03** | **.12** |
| **TGP×FGA** |  |  |  |  |  |  | **0.02** | **.02** | **.34** | **0.02** | **.02** | **.38** | **0.01** | **.02** | **.57** |
| **FGA×unemployment** |  |  |  |  |  |  |  |  |  | **0.06^+^** | **.03** | **.05** | **0.06*** | **.03** | **.03** |
| **TGP×unemployment** |  |  |  |  |  |  |  |  |  | **−0.04** | **.03** | **.16** | **−0.04** | **.03** | **.15** |
| **TGP×FGA×unemployment** |  |  |  |  |  |  |  |  |  |  |  |  | **−0.04** | **.03** | **.13** |
| df | 7, 1467 | | | 3, 1464 | | | 1, 1463 | | | 2, 1461 | | | 1, 1460 | | |
| F-value | 4.81 (*p* < .001)*** | | | 2.39 (*p* = .07)^+^ | | | 0.93 (*p* = .34) | | | 2.51 (*p* = .08) ^+^ | | | 2.27 (*p* = .13) | | |
| R² | .02 | | | .03 | | | .03 | | | .03 | | | .03 | | |
| Adjusted R² | .02 | | | .02 | | | .02 | | | .02 | | | .02 | | |

*** *p* < .001, ** *p* < .01, * *p* < .05, ^+^ *p* < .10

**Table C3**

*Multiple Regression Models after Listwise Deletion: Effects on DV3_deviance*

| Predictor | Model 1 | | | Model 2 | | | Model 3 | | | Model 4 | | | Model 5 | | |
| --- | --- | --- | --- | --- | --- | --- | --- | --- | --- | --- | --- | --- | --- | --- | --- |
|  | *β* | *SE* | *p* | *β* | *SE* | *p* | *β* | *SE* | *p* | *β* | *SE* | *p* | *β* | *SE* | *p* |
| Intercept | −0.42^+^ | .25 | .10 | −0.42^+^ | .25 | .10 | −0.43^+^ | .25 | .09 | −0.43^+^ | .25 | .09 | −0.43 ^+^ | .25 | .10 |
| Leaving certificate | −0.32 | .20 | .10 | −0.31 | .20 | .12 | −0.31 | .20 | .12 | −0.31 | .20 | .12 | 0.31 | .20 | .12 |
| Aspired type of VET | 0.44* | .17 | .01 | 0.43* | .17 | .01 | 0.43* | .17 | .01 | 0.43* | .17 | .01 | 0.43* | .17 | .01 |
| Wave | −0.92*** | .17 | < .001 | −0.91*** | .17 | < .001 | −0.91*** | .17 | < .001 | −0.91*** | .17 | < .001 | −0.91*** | .17 | < .001 |
| Migration background | 0.06 | .17 | .71 | 0.05 | .17 | .77 | 0.05 | .17 | .77 | 0.05 | .17 | .77 | 0.05 | .17 | .77 |
| Final grade | 0.24*** | .06 | < .001 | 0.26*** | .06 | < .001 | 0.26*** | .06 | < .001 | 0.26*** | .06 | < .001 | 0.26*** | .06 | < .001 |
| SES | 0.16* | .06 | .01 | 0.17** | .06 | .004 | 0.17** | .06 | .004 | 0.17** | .06 | .004 | 0.17** | .06 | .004 |
| Sex | −0.07 | .12 | .56 | −0.07 | .12 | .59 | −0.07 | .12 | .60 | −0.07 | .12 | .60 | −0.07 | .12 | .60 |
| **Unemployment** |  |  |  | **−0.00** | **.06** | **.99** | **−0.00** | **.06** | **1.00** | **0.00** | **.06** | **.99** | **−0.00** | **.06** | **.98** |
| **TGP** |  |  |  | **0.09** | **.06** | **.15** | **0.09** | **.06** | **.15** | **0.09** | **.06** | **.15** | **0.09** | **.06** | **.15** |
| **FGA** |  |  |  | **0.01** | **.06** | **.93** | **0.00** | **.06** | **.96** | **0.00** | **.06** | **.96** | **0.00** | **.06** | **.97** |
| **TGP×FGA** |  |  |  |  |  |  | **0.01** | **.05** | **.79** | **0.01** | **.05** | **.78** | **0.01** | **.05** | **.77** |
| **FGA×unemployment** |  |  |  |  |  |  |  |  |  | **−0.01** | **.06** | **.89** | **−0.01** | **.06** | **.88** |
| **TGP×unemployment** |  |  |  |  |  |  |  |  |  | **−0.00** | **.06** | **.98** | **−0.00** | **.06** | **.98** |
| **TGP×FGA×unemployment** |  |  |  |  |  |  |  |  |  |  |  |  | **0.01** | **.05** | **.90** |
| df | 7, 1379 | | | 3, 1376 | | | 1, 1375 | | | 2, 1373 | | | 1, 1373 | | |
| F-value | 8.52 (*p* < .001)*** | | | 0.80 (*p* = .49) | | | 0.07 (*p* = .79) | | | 0.01 (*p* = .99) | | | 0.02 (*p* = .90) | | |
| R² | .04 | | | .04 | | | .04 | | | .04 | | | .04 | | |
| Adjusted R² | .03 | | | .03 | | | .03 | | | .03 | | | .02 | | |

*** *p* < .001, ** *p* < .01, * *p* < .05, ^+^ *p* < .10

**Table C4**

*Multiple Regression Models after Listwise Deletion: Effects on DV4_satisfaction*

| Predictor | Model 1 | | | Model 2 | | | Model 3 | | | Model 4 | | | Model 5 | | |
| --- | --- | --- | --- | --- | --- | --- | --- | --- | --- | --- | --- | --- | --- | --- | --- |
|  | *β* | *SE* | *p* | *β* | *SE* | *p* | *β* | *SE* | *p* | *β* | *SE* | *p* | *β* | *SE* | *p* |
| Intercept | 0.11 | .12 | .36 | 0.13 | .12 | .32 | 0.11 | .12 | .36 | 0.11 | .12 | .37 | 0.11 | .12 | .37 |
| Leaving certificate | −0.03 | .10 | .75 | −0.03 | .10 | .73 | −0.03 | .10 | .75 | −0.03 | .10 | .77 | −0.03 | .10 | .76 |
| Aspired type of VET | 0.01 | .08 | .91 | 0.00 | .08 | .99 | −0.00 | .08 | .98 | −0.00 | .08 | .99 | 0.00 | .08 | 1.00 |
| Wave | −0.03 | .08 | .70 | −0.04 | .08 | .64 | −0.04 | .08 | .65 | −0.04 | .08 | .65 | −0.03 | .08 | .65 |
| Migration background | −0.17^+^ | .09 | .07 | −0.20* | .09 | .03 | −0.19* | .09 | .04 | −0.19* | .09 | .04 | −0.19* | .09 | .04 |
| Final grade | −0.04 | .03 | .21 | −0.03 | .03 | .36 | −0.03 | .03 | .33 | −0.03 | .03 | .33 | −0.03 | .03 | .33 |
| SES | −0.06^+^ | .03 | .05 | −0.06* | .03 | .04 | −0.05^+^ | .03 | .05 | −0.05^+^ | .03 | .05 | −0.05^+^ | .03 | .05 |
| Sex | −0.16* | .06 | .01 | −0.15* | .06 | .01 | −0.15* | .06 | .01 | −0.15* | .06 | .01 | −0.15* | .06 | .01 |
| **Unemployment** |  |  |  | **−0.04** | **.03** | **.13** | **−0.04** | **.03** | **.13** | **−0.04** | **.03** | **.14** | **−0.04** | **.03** | **.19** |
| **TGP** |  |  |  | **0.06*** | **.03** | **.04** | **0.07*** | **.03** | **.03** | **0.06*** | **.03** | **.03** | **0.07*** | **.03** | **.03** |
| **FGA** |  |  |  | **0.04** | **.03** | **.18** | **0.03** | **.03** | **.24** | **0.04** | **.03** | **.23** | **0.04** | **.03** | **.22** |
| **TGP×FGA** |  |  |  |  |  |  | **0.03** | **.02** | **.15** | **0.03** | **.02** | **.15** | **0.03** | **.02** | **.18** |
| **FGA×unemployment** |  |  |  |  |  |  |  |  |  | **0.02** | **.03** | **.49** | **0.02** | **.03** | **.44** |
| **TGP×unemployment** |  |  |  |  |  |  |  |  |  | **−0.03** | **.03** | **.27** | **−0.03** | **.03** | **.27** |
| **TGP×FGA×unemployment** |  |  |  |  |  |  |  |  |  |  |  |  | **−0.01** | **.02** | **.63** |
| df | 7, 1367 | | | 3, 1364 | | | 1, 1363 | | | 2, 1361 | | | 1, 1360 | | |
| F-value | 2.39 (*p* = .02)* | | | 3.42 (*p* = .02)* | | | 2.04 (*p* = .15) | | | 0.66 (*p* = .52) | | | 0.23 (*p* = .63) | | |
| R² | .01 | | | .02 | | | .02 | | | .02 | | | .02 | | |
| Adjusted R² | .01 | | | .01 | | | .01 | | | .01 | | | .01 | | |

*** *p* < .001, ** *p* < .01, * *p* < .05, ^+^ *p* < .10

Reference:

StataCorp. 2021. Stata Statistical Software: Release 17. StataCorp LLC.
